# Nanoporous ultra-high-entropy alloys containing fourteen elements for water splitting electrocatalysis†

**DOI:** 10.1039/d1sc01981c

**Published:** 2021-08-20

**Authors:** Ze-Xing Cai, Hiromi Goou, Yoshikazu Ito, Tomoharu Tokunaga, Masahiro Miyauchi, Hideki Abe, Takeshi Fujita

**Affiliations:** School of Environmental Science and Engineering, Kochi University of Technology 185 Miyanokuchi, Tosayamada Kami City Kochi 782-8502 Japan fujita.takeshi@kochi-tech.ac.jp; Institute of Applied Physics, Graduate School of Pure and Applied Sciences, University of Tsukuba Tsukuba 305-8573 Japan; Institute of Materials and Systems for Sustainability, Nagoya University Nagoya 464-8603 Japan; Tokyo Institute of Technology 2-12-1 Ookayama, Meguro-ku Tokyo 152-8552 Japan; National Institute for Materials Science 1-1 Namiki, Tsukuba Ibaraki 305-0044 Japan

## Abstract

High-entropy alloys (HEAs) are near-equimolar alloys comprising five or more elements. In recent years, catalysis using HEAs has attracted considerable attention across various fields. Herein, we demonstrate the facile synthesis of nanoporous ultra-high-entropy alloys (np-UHEAs) with hierarchical porosity *via* dealloying. These np-UHEAs contain up to 14 elements, namely, Al, Ag, Au, Co, Cu, Fe, Ir, Mo, Ni, Pd, Pt, Rh, Ru, and Ti. Furthermore, they exhibit high catalytic activities and electrochemical stabilities in the hydrogen evolution reaction (HER) and oxygen evolution reaction (OER) in acidic media, superior to that of commercial Pt/graphene and IrO_2_ catalysts. Our results offer valuable insights for the selection of elements as catalysts for various applications.

## Abstract 2

Nanoporous ultra-high-entropy alloys containing 14 elements (Al, Ag, Au, Co, Cu, Fe, Ir, Mo, Ni, Pd, Pt, Rh, Ru, and Ti) were obtained by dealloying. The products showed excellent electrocatalytic performance for water splitting in acidic media.

## Graphical abstract

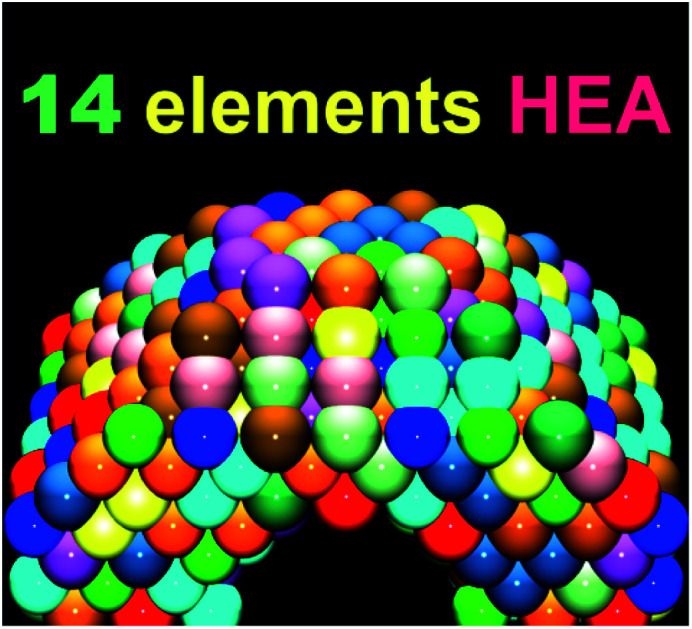

## Introduction

Developing highly effective water-splitting catalysts is essential for addressing the worsening energy crisis and long-term environmental pollution. In recent years, multimetallic compounds have been increasingly developed as effective catalysts for electrochemical overall water-splitting, owing to the synergistic effects between various components.^1–3^ The distinct components in multimetallic compounds can facilitate the adsorption/desorption of various ions or regulate charge transfer, thus promoting the overall reaction.^4^ In addition, multimetallic compounds are amenable to structure optimization, crucial for maximizing the exposure of active sites and improving long-term stability.^5,6^ High-entropy alloys (HEAs), first reported in 2004, are solid solutions composed of at least five metallic elements with atomic concentrations ranging from 5% to 35%.^7–10^ HEAs have been drawing increasing attention from both theoretical and experimental perspectives as the high degree of synergy therein leads to high entropy, lattice distortion, sluggish diffusion, and the cocktail effect. The salient feature of HEAs is their high configurational entropy, which allows the resultant materials to exist in a stable solid-solution phase, and is beneficial for enhancing the hardness and strength, as well as resistance to wear, oxidation, corrosion, and other degradation factors.^9–11^ While the mechanical properties of HEAs have been extensively investigated, their application as functional materials, such as catalysts, has only recently been probed.^12,13^ The active surfaces of HEAs consist of millions of different possible atomic arrangements, and some surface sites may have optimal properties that can overcome the limitations of current catalysts. Another disadvantage of prevailing catalytic processes is that they rely heavily on precious metal catalysts with scarce resources. For example, green energy conversion reactions entail water electrolysis, wherein precious Pt and noble-metal-based compounds, for instance, oxides such as RuO_2_ and IrO_2_, are still state-of-the-art catalysts for the hydrogen evolution reaction (HER) and oxygen evolution reaction (OER). Therefore, there is considerable motivation to combine precious metals with as many cheaper metals as possible to reduce the usage of precious metals with the aim of improving activity. To date, the maximum number of constituent elements in HEAs remains an open question, and it is unknown whether a nanostructured HEA containing diverse atoms would deliver an omnipotent catalyst owing to its elemental diversity and functionality. In an fcc structure, the number of nearest neighboring atoms is 12, and assuming that the maximum number of constituent elements in HEAs is more than 13 (12 + central atom), each nearest neighboring atom could be distinct. These special local atomic environments are considered to be in an ultra-high-entropy state.

Rational optimization of the composition and geometric structure of HEAs is essential for improving their catalytic activity; however, to the best of our knowledge, the development of facile and controlled syntheses of nanoscale HEA-based catalysts is still in its infancy. Several techniques have been reported for constructing nanostructured HEAs intended for heterogeneous catalysis, including the carbothermal shock technique,^14,15^ mechanical alloying,^16^ fast moving bed pyrolysis,^17^ chemical synthesis,^18–20^ and dealloying.^21–24^ Among these methods, dealloying is an electrochemical process commonly employed for the selective dissolution of less stable elements from precursor alloys. This method allows for the retention of metals to form a bi-continuous metal-and-void hierarchical porous structure. Recently, this technique has been applied for tailoring metallic materials with designed constituents and unique pore structures, which feature various advantageous properties for catalysis, including a high specific surface area, low density, reduced path lengths for mass and charge transfer, and efficient material utilization.^25,26^

Herein, we describe the synthesis of np-UHEAs containing up to 14 elements, namely, Al, Ag, Au, Co, Cu, Fe, Ir, Mo, Ni, Pd, Pt, Rh, Ru, and Ti, *via* one-step dealloying. The elements are among the main transition elements and noble metals frequently used in catalysis,^27–30^ and Al is the sacrificial element used in dealloying. The estimated mixed configurational entropy (*S*) of a sample comprising 14 elements exceeds 2.5 *R* (*S* ≥ 2.5*R*), where *S* is defined as 
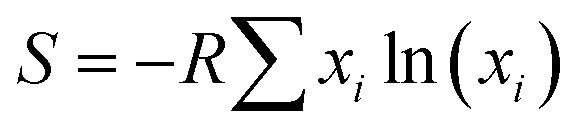
, *R* is the molar gas constant, and *x*_*i*_ is the mole fraction of the elemental component. Microstructural characterization of np-UHEAs indicated a hierarchical nanoporous structure with uniform distribution of each element and high surface areas (>50 m^2^ g^−1^), making them suitable for electrocatalysis. The np-UHEAs exhibited high catalytic activities for the HER and OER in acidic solution as well as electrochemical stability, outperforming commercial 20 wt% Pt/graphene (Pt/G) HER and IrO_2_ OER catalysts.

## Results and discussion

### Fabrication

**Fig. 1:**
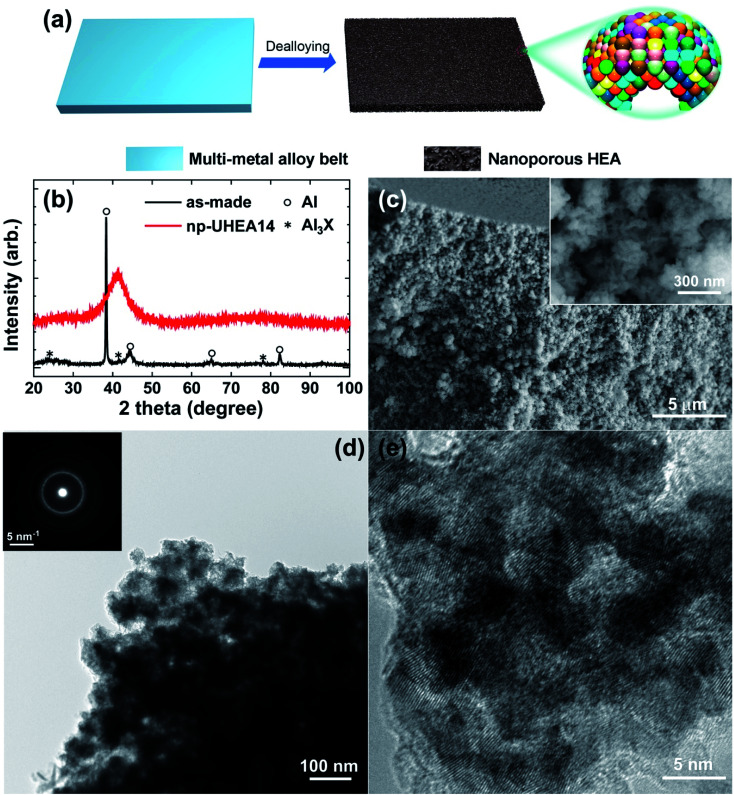
(a) Schematic illustration of the preparation of nanoporous ultra-high-entropy alloys (np-UHEAs). (b) X-ray diffraction (XRD) patterns of as-made precursor alloy Al_87_Ag_1_Au_1_Co_1_Cu_1_Fe_1_Ir_1_Mo_1_Ni_1_Pd_1_Pt_1_Rh_1_Ru_1_Ti_1_ quenched by melt spinning to obtain ribbons and np-UHEA14 after dealloying. (c) Scanning electron microscopy (SEM) images of the cross-section regions of np-UHEA14. (d) Low-magnification transmission electron microscopy (TEM) image of np-UHEA14 showing nanoporosity of ∼50 nm and the corresponding selected-area diffraction pattern showing the fcc structure. (e) High-resolution TEM image showing small nanopores ∼5 nm in size and randomly oriented nanocrystallinity.

All experimental procedures are described in the Experimental section. Briefly, multi-metal alloy ingots with atomic ratios of Al_87_Ag_1_Au_1_Co_1_Cu_1_Fe_1_Ir_1_Mo_1_Ni_1_Pd_1_Pt_1_Rh_1_Ru_1_Ti_1_, Al_88_Ag_1_Au_1_Co_1_Cu_1_Fe_1_Ir_1_Mo_1_Ni_1_Pd_1_Pt_1_Rh_1_Ru_1_, and Al_89_Ag_1_Au_1_Co_1_Cu_1_Fe_1_Ir_1_Ni_1_Pd_1_Pt_1_Rh_1_Ru_1_ (%) were prepared by arc melting pure commercially available metal powders (>99.9%) in an inert atmosphere. Thus, the 12-element alloy contained Fe, Ru (Group VIII), Co, Rh, Ir (Group IX), Ni, Pd, Pt (Group X), Cu, Ag, Au (Group XI), and Al (Group XIII) elements, while the 14-element alloy additionally contained Mo (Group VI), and Ti (Group III), and the 13-element alloy additionally contained Mo. All alloy ribbons with thicknesses of ∼30–40 μm and widths of 2 mm were produced by re-melting the ingots on the cold surface of a spinning copper roller. Np-UHEA ribbons with varying compositions were prepared by chemical dealloying in 0.5 M NaOH at room temperature for 3 h (Fig. 1(a)). The UHEAs obtained by dealloying the 12-, 13-, and 14-element alloys are denoted as np-UHEA12, np-UHEA13, and np-UHEA14, respectively. The etched ribbons were washed several times with deionized water and then dried under ambient conditions.

First, we tested two different quenching techniques, namely, gas atomization and melt spinning. Fig. S1(a)† indicates that a higher number of intermetallic counterparts (Al_3_X) were formed using the gas atomization technique because the higher cooling rate employed in melt spinning suppressed their formation. The 0.5% sample produced *via* melt spinning contained a higher degree of Al_3_X compared to the 1% sample, while the latter was more amenable to further processing, as shown in Fig. S1(b).† Similar to metallic glass formation based on the confusion principle,^31,32^ competitive thermodynamics between the Al fcc phase and intermetallic counterparts during cooling needs to be considered.

### Characterization

Fig. 1(b) shows the X-ray diffraction (XRD) patterns of the as-made precursor alloy Al_87_Ag_1_Au_1_Co_1_Cu_1_Fe_1_Ir_1_Mo_1_Ni_1_Pd_1_Pt_1_Rh_1_Ru_1_Ti_1_ quenched using melt spinning to obtain the ribbon sample, and that of np-UHEA14 after dealloying. Only the pure Al phase was detected in the former due to the high atomic percentage of Al in the precursor. The formation of intermetallics (AlX_3_) was also suppressed by rapid quenching, suggesting the uniform distribution of other elements in the Al matrix. After dealloying, only a broad peak at approximately 40.5° (2*θ*) was observed, which was indexed to the (111) diffraction of the fcc structure. Furthermore, the broadness of the peak also implied that nanometer-sized crystals dominated the sample and that the 14 metal species were uniformly distributed in one solid phase comprising varying atomic radii. Similar results were obtained for np-UHEA12 and np-UHEA13 (Fig. S2†). The SEM image of np-UHEA14 (Fig. 1(c)) shows a porous structure with large pore channels (∼50–100 nm). Cross-section images revealed that the pore channels propagated through the entire np-UHEA14 sample; the same was observed for np-UHEA12 and np-UHEA13 (Fig. S3†). Fig. 1(d) depicts a low-magnification TEM image of np-UHEA14 with a nanoporosity of ∼50 nm, and the corresponding selected-area diffraction pattern reveals nanometer-sized crystals with a typical fcc structure. The high-resolution TEM image in Fig. 1(e) shows small nanopores (∼2–5 nm) and randomly oriented nanocrystallinity. Therefore, np-UHEA14 displayed hierarchical nanoporosities of ∼2–5 nm and ∼50 nm. These hierarchies were confirmed by acquiring nitrogen adsorption and desorption isotherms to determine Barrett–Joyner–Halenda (BJH) pore size distributions for np-UHEA14, np-UHEA12, and np-UHEA13 (Fig. S4†). The hierarchical nanoporosities arise from the fast and slow dissolution kinetics of Al during dealloying corresponding to the ∼50 nm and ∼2–5 nm pore sizes, respectively.^33^ The Brunauer–Emmett–Teller (BET) surface areas of np-UHEA12, np-UHEA13, and np-UHEA14 were 56.8, 48.2, and 55.2 m^2^ g^−1^, respectively.

**Fig. 2:**
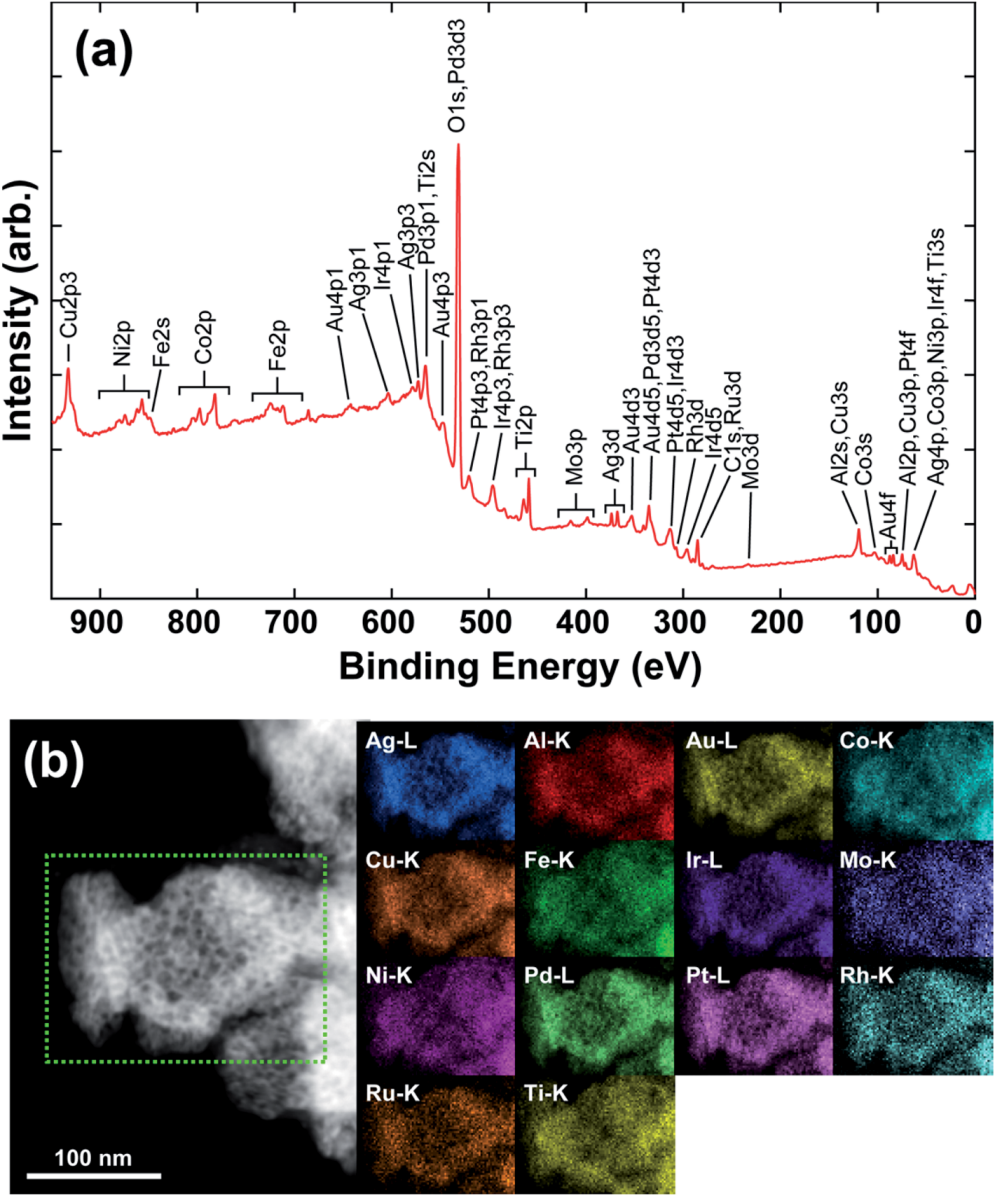
(a) Complete XPS spectrum of np-UHEA14 in the 0−950 eV region showing the existence of 14 elements. (b) High-angle dark field-STEM image of np-UHEA14 and the corresponding EDS maps for each characteristic peak.

Fig. 2(a) shows the complete X-ray photoemission spectroscopy (XPS) results for np-UHEA14 in the range of 0–950 eV, revealing the chemical state of each constituent element. The selected spectrum and deconvoluted waves for each elemental state are displayed in Fig. S5.† Al and the transition metals (Co, Cu, Fe, Mo, Ni, and Ti) were predominantly in their oxidation state owing to their high reactivity, while the noble metals (Ag, Au, Ir, Pd, Pt, Rh, and Ru) were present in their principal metallic state. It has been demonstrated that non-noble metals are readily oxidized to form oxides/hydroxides during dealloying in alkaline aqueous solutions, while noble metals remain relatively stable. Thus, our results are consistent with previously reported findings.^34,35^ The combination of transition metal oxidation states and noble metal metallic states has been shown to be beneficial for HER and OER activity.^18,22^ We confirmed that each characteristic X-ray line could be distinguished in the elemental map (Fig. S6†). Fig. 2(b) shows the scanning transmission electron microscopy (STEM) image and the corresponding energy-dispersive X-ray spectrometry (EDS) map, wherein a uniform distribution of elements is evident. Uniform distribution of elements was also confirmed in np-UHEA12 and np-UHEA13, as shown in Fig. S7(a) and (b).†

### HER and OER performances

**Fig. 3:**
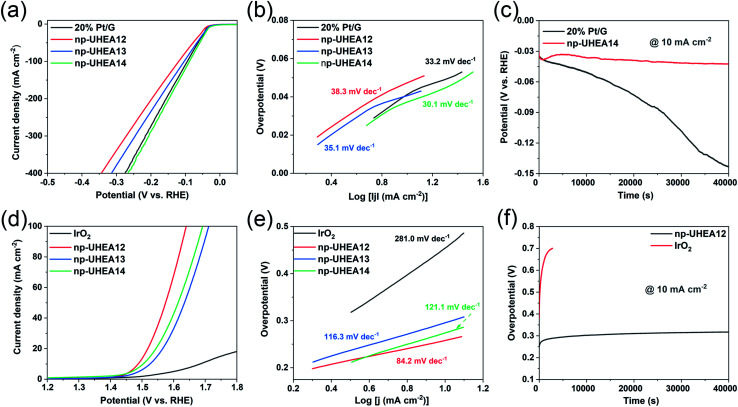
(a) Polarization curves and (b) the corresponding Tafel plots of commercial 20% Pt/G, np-UHEA12, np-UHEA13, and np-UHEA14 electrodes for the HER in 0.5 M H_2_SO_4_. (c) Chronopotentiometry curves (*V*–*t*) of the np-UHEA14 electrode and commercial 20% Pt/G electrode in 0.5 M H_2_SO_4_ at a constant current density of 10 mA cm^−2^. (d) Polarization curves and (e) the corresponding Tafel plots of commercial IrO_2_, np-UHEA12, np-UHEA13, and np-UHEA14 electrodes for the OER in 0.5 M H_2_SO_4_. (f) Chronopotentiometry responses (*V*–*t*) collected for the np-UHEA12 electrode and commercial IrO_2_ at a current density of 10 mA cm^−2^ over 40 000 s in 0.5 M H_2_SO_4_.

The electrocatalytic HER and OER performances of the as-prepared np-UHEAs were evaluated in 0.5 M sulfuric acid (H_2_SO_4_) using a standard three-electrode system. For comparison, a commercially available Pt on graphene (Pt/G, 20 wt%) benchmark electrocatalyst was also studied under the same conditions. Prior to the linear sweep voltammetry (LSV) test, a stable state of the np-UHEA catalysts was achieved by several cyclic voltammetry (CV) cycles at a sweep rate of 100 mV s^−1^ between 1.1 V and 1.6 V *versus* reversible hydrogen electrode (RHE) in the electrolyte. The CV cycling process can help remove the unstable metal species on the surface and expose the noble metal rich surface.^21^ The HER activities of np-UHEA14 before and after CV cycling were compared, and a prominent increase in the HER activity was observed (Fig. S8†). Therefore, the oxidation of the transition metals on the surface results in surface passivation for the HER. The LSV curves of the np-UHEAs and Pt/G catalysts, shown in Fig. 3(a), indicate that the np-UHEA14 catalyst achieved a current density of 10 mA cm^−2^ at an overpotential of 32 mV, which was lower than that of the Pt/G (36 mV), np-UHEA13 (36 mV), and np-UHEA12 (42 mV) catalysts. The hydrogen evolution activity of np-UHEA alloys was also evaluated by comparison of their mass activity with that of Pt at −0.1 V *vs.* RHE; the mass activities of np-UHEA14, np-UHEA13, and np-UHEA12 were 2.44, 1.70, and 1.32 A mg_Pt_^−1^, respectively, while that of Pt/G was only 1.08 A mg_Pt_^−1^. Although it is difficult to interpret this highly complex system, the above-listed results clearly demonstrate that the incorporation of Mo and Ti promotes HER activity as a result of electronic modification.^29,30^ The corresponding Tafel slopes are presented in Fig. 3(b); the superior catalytic activity of np-UHEA14 was further illustrated by its Tafel slope being the smallest (30.1 mV dec^−1^) compared with those of np-UHEA12 (38.3 mV dec^−1^), np-UHEA13 (35.1 mV dec^−1^), and commercial Pt/G (33.2 mV dec^−1^). Thus, the Tafel plots suggest that the HER kinetics of np-UHEA14 were the fastest. Furthermore, it can be inferred from the Tafel slope that the np-UHEA14-catalyzed HER process proceeds through the Volmer–Tafel mechanism. The relatively low overpotential at 10 mA cm^−2^ in 0.5 M H_2_SO_4_ electrolyte and the low Tafel slope suggested that np-UHEA14 required less energy for catalytic hydrogen production than the other tested catalysts. The Nyquist plots (Fig. S9(a)†) show that np-UHEA14 exhibited the lowest charge-transfer impedance at 0.0 V *vs.* RHE, suggesting rapid electron transport, which leads to a marked acceleration of HER kinetics. The long-term stabilities of the np-UHEA14 and Pt/G electrocatalysts were further evaluated and compared by conducting chronopotentiometry measurements at a current density of 10 mA cm^−2^. Notably, np-UHEA14 showed only a slight increase in potential after 10 h, while Pt/G showed a rapid potential increase within 10 h, indicating that np-UHEA14 was highly stable (Fig. 3(c)). XRD measurements were performed after the stability test, and no obvious phase transformations or phase separation were detected in the patterns (Fig. S10(a)†). The porous morphology was almost completely maintained, as shown in the SEM (Fig. S11(a) and (b)†) and TEM (Fig. S12(a) and (b)†) images. Furthermore, the EDX results (Fig. S12(c)†) indicated that the proportion and distribution of all the surface elements remained unchanged after the stability test.

The OER occurring at the anode is another critical half reaction in overall water splitting. The OER proceeds through a complex four-step proton-coupled electron transfer process and is regarded as the thermodynamic and kinetic rate-limiting reaction.^36,37^ The electrocatalytic OER performance of the np-UHEA catalysts in acidic environments was also evaluated, and a commercial IrO_2_ catalyst was used for comparison. In 0.5 M H_2_SO_4_, the np-UHEA12 catalyst gave rise to an OER overpotential of 258 mV at 10 mA cm^−2^ (Fig. 3(d)) and a Tafel slope of 84.2 mV dec^−1^ (Fig. 3(e)), both being significantly lower than those of np-UHEA13 (294 mV, 116.3 mV dec^−1^), np-UHEA14 (274 mV, 121.1 mV dec^−1^), and commercial IrO_2_ (454 mV, 281.0 mV dec^−1^), suggesting the superiority of np-UHEA12 as an oxygen production catalyst under acidic conditions. When the impedance was measured at 1.5 V *vs.* RHE, the Nyquist plot of np-UHEA12 exhibited the lowest charge-transfer impedance (Fig. S9(b)†). This result indicates that np-UHEA12 displayed the fastest catalytic kinetics for the dissociation of water and the OER. Developing highly durable electrocatalysts for the continuous OER in acidic media is challenging. Thus, the stabilities of np-UHEA12 and commercial IrO_2_ were evaluated; the chronopotentiometric curve of IrO_2_ shown in Fig. 3(f) reveals an appreciable potential increase within 1 h of oxygen evolution, while that of np-UHEA12 confirms its excellent long-term stability for the acidic OER (10 h). Similarly, the XRD (Fig. S10(b)†), SEM (Fig. S11(c) and (d)†), and TEM (Fig. S13†) results also confirmed that the crystal phase, composition, overall morphology, and porous structure of the catalyst were well maintained after long-term OER catalysis in the acidic electrolyte, apart from a slight decrease in the proportion of Al and a slight roughening of the surface of the nanoplates as compared to the pristine nanoplates, possibly caused by the dissolution of Al in the acidic medium. From the above-discussed results, it can be concluded that the np-UHEAs exhibited excellent activity and stability in both OER and HER catalyses, attributable to their favorable structural and compositional characteristics, such as their unique porous structure, large surface area, and evenly distributed elements.

It is well known that the electrochemically active surface area (ECSA) is proportional to the double-layer capacitance (*C*_dl_), which can be evaluated by obtaining cyclic voltammograms at various scan rates (Fig. S14†).^38,39^ The calculated *C*_dl_ of np-UHEA14 was 9.0 mF cm^−2^, which was larger than those of np-UHEA13 (6.7 mF cm^−2^) and np-UHEA12 (4.7 mF cm^−2^), revealing that a larger proportion of active sites were available in the np-UHEA14 catalyst (Fig. S14(d)†). Thus, evidently np-UHEA14 possesses the largest ECSA and offers more abundant active catalytic sites than np-UHEA13 and np-UHEA12. The underpotential deposition of copper (Cu-UPD) on noble metals is considered an ideal method for characterizing the corresponding active sites. Therefore, Cu-UPD was applied to evaluate the ECSAs of all the samples (Fig. S15 and S16†). According to the theoretical specific surface area calculations, the ECSA_Cu-upd_ values of np-UHEA12, np-UHEA13, np-UHEA14, Pt/G, and IrO_2_ were found to be 32.0, 18.8, 27.4, 11.9, and 3.1 m^2^ g^−1^, respectively (Fig. S17†), indicating significantly enhanced exposure and atomic utilization of noble metals in the nanoporous structure compared to that in commercial Pt/G and IrO_2_. The higher ECSA_Cu-upd_ of np-UHEA12 compared to those of np-UHEA13 and np-UHEA14 possibly stems from its higher noble metal content and differences in pore structure.

The experimental results show that the addition of Mo and Ti to np-UHEA12 resulted in a slight decrease in its OER activity, and an increase in the HER activity. It is believed that the efficient catalytic activity of HEA systems is largely attributable to electronic effects and the synergistic effect among the various elements.^22,40,41^ However, the synergistic effect arising from 12 or 14 elements in one solid phase is too complex to define owing to the sheer number of element combinations, and it is near-impossible to precisely predict or explain the considerable effect through theoretical and/or experimental methods. Therefore, the research system needs to be simplified. Among the 14 elements, the principal OER active sites are considered to be contributed by Ru and Ir, while the HER active sites are predominantly provided by Ru, Ir, and Pt. Therefore, for the OER activity, we only considered the synergies between Mo/Ti and Ru/Ir, and for the HER, we only studied the synergies between Mo/Ti and Ru/Ir/Pt. In the case of the OER, the reaction is relatively more complex because of the transfer of four electrons *via* several proton/electron-coupled procedures within one electrocatalytic cycle. Therefore, enhancing the OER activity of Ru/Ir-based materials by introducing Mo and Ti is generally met with more restrictive conditions. For example, several reports have confirmed that anatase-type TiO_2_–IrO_2_ solid solutions possess more active Ir catalytic sites for the OER than IrO_2_, while the same was not observed for their rutile-type counterparts.^42–44^ Similarly, Peron *et al.* found that Mo does not affect the intrinsic activity of Ir in IrMo-mixed oxides.^45^ However, in some cases, it is thought that the covalency of the Ir–O bond increases as a result of introducing Mo, thus resulting in superior OER activity.^21,46^ Therefore, it can be qualitatively concluded that the doping of Mo and Ti in np-UHEA12 reduces the content of Ru and Ir, causing a decrease in the OER activity due to the lower number of active sites. The synergistic effect between Ti/Mo and Ir can enhance or decrease the OER activity to a certain extent due to the existence of various combinations, given the overall result of a slight decrease in OER activity. In the case of the HER, the reaction only involves the adsorption/desorption of hydrogen (*H/H_2_), and hydrogen binding energy (HBE) is generally considered as the sole descriptor for evaluating the HER activity. Numerous previous studies have confirmed that alloying Ru, Ir, or Pt with Mo and Ti can significantly increase their intrinsic HER activity by modulating the electronic structure and H-bond strength.^47–50^ Therefore, the introduction of Mo and Ti expectedly increased the HER activity.

**Fig. 4:**
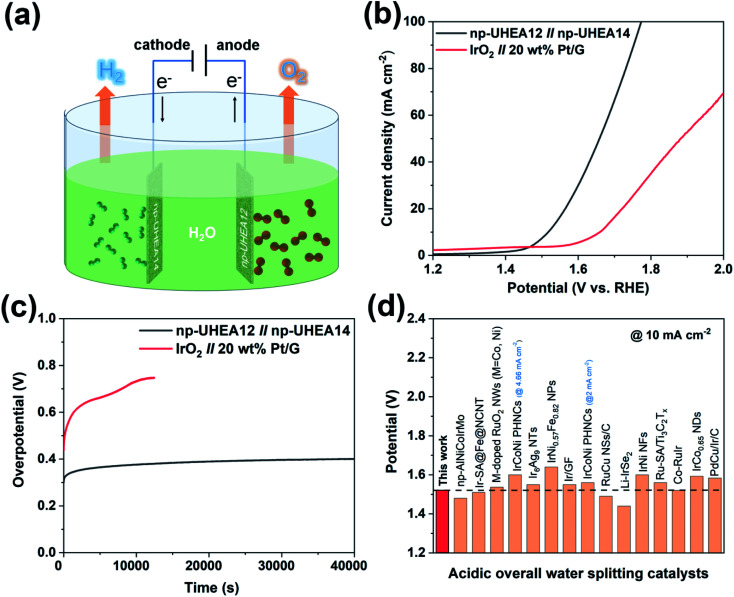
(a) Schematic illustration of overall water splitting using np-UHEAs as catalysts. (b) Polarization curves of overall water splitting catalyzed by the np-UHEA12||np-UHEA14 couple and IrO_2_||Pt/G couple at a scan rate of 5 mV s^−1^ in 0.5 M H_2_SO_4_. (c) Chronopotentiometric curves of np-UHEA12||np-UHEA14 couple and IrO_2_||Pt/G couple under a current density of 10 mA cm^−2^. (d) Cell voltage values of notable acidic overall water spitting catalysts reported in the literature at a current density of 10 mA cm^−2^ (corresponding references are shown in Table S2†).

Taking advantage of these unique properties, the nanoporous catalysts were further utilized to assemble overall water splitting devices, where np-UHEA12 was used as the anodic electrode for the OER and np-UHEA14 was employed as the cathodic electrode for the HER. IrO_2_||Pt/G, composed of commercial Pt/G as the HER catalyst and commercial IrO_2_ as the OER catalyst were selected for comparison. The polarization curves of np-UHEA12||np-UHEA14 and IrO_2_||Pt/G for overall water splitting were obtained in 0.5 M H_2_SO_4_ using a two-electrode setup (Fig. 4(a)). Remarkably, the np-UHEA12||np-UHEA14 couple showed excellent overall activity for water splitting, whereby a cell voltage of only 1.53 V was required, whereas the IrO_2_||Pt/G couple required 1.66 V at a splitting current density of 10 mA cm^−2^ (Fig. 4(c)). In addition, the long-term durability of the np-UHEA12||np-UHEA14 couple and IrO_2_||Pt/G for water splitting was also studied employing chronopotentiometry (Fig. 4(c)). The results indicate that np-UHEA12||np-UHEA14 outperforms the majority of recently reported HEA-based catalysts and other bifunctional catalysts for water splitting (Fig. 4(d), Tables S1 and S2†). The np-UHEA12||np-UHEA14 couple showed a ∼100 mV increase in potential after continuous electrolysis for 40 000 s at 10 mA cm^−2^, while a significant increase in the potential of the IrO_2_||Pt/G couple was observed after ∼10 000 s. Such remarkable activity demonstrates the potential applicability of np-UHEAs for energy-efficient overall acidic water splitting.

## Conclusions

In this study, the dealloying method was applied as a facile synthetic route to np-UHEAs containing up to 14 elements (Al, Ag, Au, Co, Cu, Fe, Ir, Mo, Ni, Pd, Pt, Rh, Ru, and Ti). The dealloying strategy shows great potential for the design and preparation of np-UHEAs as it can accommodate a far greater number of elements than the 14 used in this study. TEM analysis of the alloys indicated a hierarchical, nanoporous, and nanocrystalline fcc structure with uniformly distributed elements. The nanoscale porous structure introduced by the leaching of Al metal increased the number of accessible active sites on the surface and contact with the electrolyte. The np-UHEAs were employed as electrocatalysts, displaying high activity in energy-efficient overall acidic water splitting. The facile etching approach enables straightforward modification of the composition and structure of np-HEAs for various complex reactions. The universality of this alloying/dealloying strategy will be further explored to include other metal elements and various combinations. In addition to transition metal elements, non-metals, such as sulfur, phosphorus, selenium, and boron could also be introduced to prepare high-entropy sulfides, phosphides, selenides, and borides, respectively. We intend to tune the composition and structure of np-HEAs by selecting suitable transition metal elements to achieve multifunctionality or expand their applicability. This study not only provides the basis for prospecting and designing np-HEAs with diverse compositions and structures, but also provides unprecedented opportunities for element selection for fabricating an omnipotent catalyst, or an “omni-catalyst,” for various applications. In the future, material informatics may assist in further element optimization over high-dimensional composition space.^51,52^

## Experimental

### Synthesis of nanoporous ultra-high-entropy alloys (np-UHEAs)

Multi-metal (AlAgAuCoCuFeIrMoNiPdPtRhRuTi) alloy ingots with atomic ratios of Al_87_Ag_1_Au_1_Co_1_Cu_1_Fe_1_Ir_1_Mo_1_Ni_1_Pd_1_Pt_1_Rh_1_Ru_1_Ti_1_, Al_88_Ag_1_Au_1_Co_1_Cu_1_Fe_1_Ir_1_Mo_1_Ni_1_Pd_1_Pt_1_Rh_1_Ru_1_, and Al_89_Ag_1_Au_1_Co_1_Cu_1_Fe_1_Ir_1_Ni_1_Pd_1_Pt_1_Rh_1_Ru_1_ (%) were prepared by arc melting pure commercial metal powders (>99.99%) in a pure Ar atmosphere. After verifying the compositions of the ingots, alloy ribbons of ∼20 μm thickness and 2 mm width were produced by re-melting the ingots on the cold surface of a spinning copper roller at a speed of 40 m s^−1^. The np-UHEA ribbons of varying compositions were prepared by chemical dealloying in 0.5 M NaOH at room temperature for 3 h. The etched ribbons were washed several times with deionized water to remove the remaining chemical substances in the nanopore channels, and then dried under ambient conditions. For comparison, commercial 20 wt% Pt/G (No. 738549, Sigma-Aldrich, USA) and IrO_2_ (No. 097-03571, Wako, Japan) were used.

### Characterization

X-ray diffraction (XRD) patterns of the samples were obtained using a Rigaku RINT 2000 X-ray diffractometer with monochromatic Cu Kα radiation (40 kV, 40 mA). The microstructure was inspected using a Hitachi S-8020 scanning electron microscope (SEM) at an accelerating voltage of 10 kV. The microstructures of the obtained catalysts were characterized by transmission electron microscopy (TEM, JEM-2100F and JEM-ARM200F “NEO ARM,” JEOL, equipped with aberration correctors, for the image- and probe-forming lens systems, CEOS GmbH) and energy-dispersive X-ray spectrometry (EDS, JED-2300T, JEOL). TEM and scanning TEM (STEM) observations were conducted at an accelerating voltage of 200 kV. The samples were transferred onto a carbon-coated nylon mesh (No. 10395, LADD Research Industries, USA) for X-ray analysis. The Brunauer–Emmett–Teller (BET) surface areas of the samples were measured at 77 K using a BELSORP-MAX II (MicrotracBEL Japan, Inc.). Pore sizes were determined using the Barrett–Joyner–Halenda (BJH) method. Each sample was heated at 80 °C under vacuum for 12 h prior to measurement, and the mass of each sample was measured using a balance. The contents of each sample were determined using an inductively coupled plasma-optical emission spectrometer (ICP-OES, ICPS-8100, Shimadzu, Japan). Hard X-ray photoemission spectroscopy (HAXPES) was conducted using an X-ray with a photon energy of 5.95 keV at the undulator beamline BL15XU of SPring-8, Japan. The HAXPES spectra were acquired at room temperature under UHV using a hemispherical electron energy analyzer (VG SCIENTA R4000). The binding energy was referenced to the Fermi edge of the Au thin film.

### Electrocatalytic measurements

An IviumStat electrochemical workstation (IVIUM Technologies B.V., The Netherlands) equipped with a three-electrode system was used to conduct electrocatalytic measurements at room temperature. A graphite rod and an Ag/AgCl electrode were used as the counter and reference electrodes, respectively. To prepare the working electrode, np-UHEA powder (5 mg) and Nafion solution (12.5 μL) were added to 500 μL of water–ethanol solution (1 : 3 v/v), followed by ultrasonic irradiation for 2 h in ice water to form a uniform ink. An aliquot (10 μL) of the as-prepared ink was loaded onto a piece of carbon paper (2 mm × 20 mm with a loading area of 2 mm × 10 mm, loading ∼0.25 mg cm^−2^, Fuel Cell Earth, Japan) and allowed to dry under ambient conditions. The resulting carbon paper was transferred to an electrode holder for use as the working electrode. The electrolyte solutions were purged with nitrogen (purity: 99.999%) for at least 30 min prior to use. The polarization curves of the hydrogen evolution reaction (HER) and oxygen evolution reaction (OER) were measured at a scan rate of 5.0 mV s^−1^ in 0.5 M H_2_SO_4_ (pH = 0). All measured potentials were calibrated to a reversible hydrogen electrode (RHE) using the following equation: *E*_RHE_ = *E*_Ag/AgCl_ + (0.197 + 0.0591 × pH). All data were collected using the *iR* compensation. To obtain the Tafel plots, the polarization curves were plotted as the overpotential *vs.* log of current density, and the linear portions at low overpotentials were fitted to the Tafel equation (*η* = *a* + *b* log *j*, where *η* is the overpotential, *b* is the Tafel slope, *j* is the current density, and *a* is the exchange current density). Electrochemical impedance spectroscopy (EIS) measurements were conducted in potentiostatic mode in the frequency range from 1 M to 0.1 Hz under the operating voltage. Cyclic voltammetry curves in the region of 0.25–0.40 V *vs.* RHE were measured under varying scan rates. The electrochemically active surface area (ECSA) was evaluated from the slope of the plot of the charging current *versus* the scan rate, which was proportional to the double-layer capacitance, *C*_dl_. The long-term stability of the catalysts was determined using chronopotentiometry measurements.

### Electrochemical surface area (ECSA) measurements

Underpotential deposition (UPD) of copper on noble metals has proven to be an ideal method for characterizing the corresponding active sites. With this approach, the ECSA can be calculated based on the UPD copper stripping charge (*Q*_Cu_, Cu_upd_ → Cu^2+^ + 2e^−^) using the following equation:^53,54^
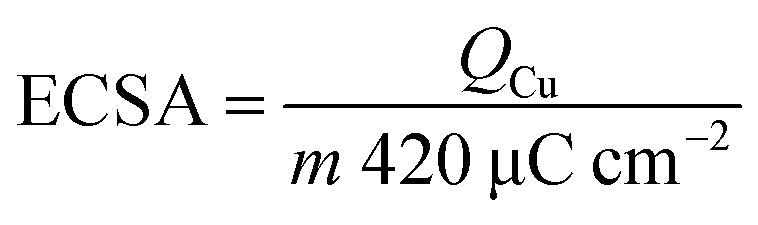


As shown in Fig. S15,† np-UHEA12 was first polarized at 0.240–0.250 V during 100 s (Fig. S15(a)†). Under polarization potentials of 0.250 and 0.248 V, two oxidation peaks were observed at ∼0.30 V and 0.55 V, corresponding to the underpotentially deposited monolayer or sub-monolayer copper. When the potential was decreased to 0.246–0.240 V, the peak at ∼0.29 V increased dramatically, which can be attributed to the oxidation of bulk copper. Therefore, a potential of 0.248 V was applied to obtain a monolayer of copper in the following test of np-UHEA12 (Fig. S15(b)†). Furthermore, np-UHEA13 (Fig. S15(c) and (d)†), np-UHEA14 (Fig. S15(e) and (f)†), commercial Pt/graphene (Fig. S16(a) and (b)†), and IrO_2_ (Fig. S16(c) and (d)†) were investigated using the same method. Based on the above information, the ECSA of np-UHEA12, np-UHEA13, np-UHEA14, commercial Pt/G, and IrO_2_ were calculated to be 32, 18.8, 27.4, 11.9, and 3.1 m^2^ g^−1^, respectively, as summarized in Fig. S17.†

## Author contributions

T. F. conceived the idea, planned and supervised the study, and designed the experiments. Z. C. and T. F. fabricated the samples. Z. C. and Y. I. performed the electrochemical measurements. T. F., H. G., and T. T. conducted the TEM/STEM/SEM characterizations. A. H. and M. M. performed the XPS analysis. Z. C., M. M, A. H., and T. F. wrote the manuscript and provided an explanation of the experiments. All authors discussed the results and gave inputs for the manuscript.

## Conflicts of interest

The authors declare no competing interests.

## Supplementary Material

SC-012-D1SC01981C-s001
