# Supplementary material for: Nanoporous ultra-high-entropy alloys containing fourteen elements for water splitting electrocatalysis
Source: Chem Sci. 2021 Aug 20;12(34):11306–15. doi: 10.1039/d1sc01981c (PMC8447928; doi:10.1039/d1sc01981c)
Supplement: SC-012-D1SC01981C-s001 [file SC-012-D1SC01981C-s001.pdf]

Electronic Supplementary Material (ESI) for Chemical Science.
This journal is © The Royal Society of Chemistry 2021

Supporting Information

Nanoporous ultra-high-entropy alloys containing fourteen elements for water splitting electrocatalysis

*Ze-Xing Cai, Hiromi Goou, Yoshikazu Ito, Tomoharu Tokunaga, Masahiro Miyauchi, Hideki Abe, Takeshi Fujita**

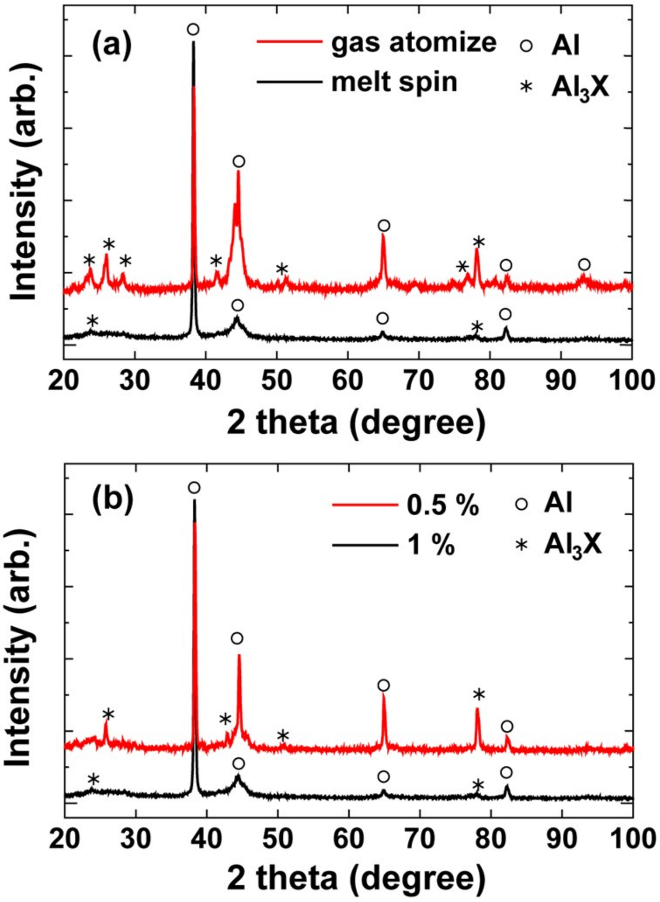

Figure S1. (a) XRD patterns of as-made precursor alloy $Al_{89}Ag_1Au_1Co_1Cu_1Fe_1Ir_1Ni_1Pd_1Pt_1Rh_1Ru_1$ quenched via gas atomization for powder form or melt spinning for ribbon form. (b) XRD patterns of as-made precursor alloys $Al_{89}Ag_1Au_1Co_1Cu_1Fe_1Ir_1Ni_1Pd_1Pt_1Rh_1Ru_{1,}$ (denoted as 1%) and $Al_{94.5}Ag_{0.5}Au_{0.5}Co_{0.5}Cu_{0.5}Fe_{0.5}Ir_{0.5}Ni_{0.5}Pd_{0.5}Pt_{0.5}Rh_{0.5}Ru_{0.5}$ (denoted as 0.5%), quenched using the melt spinning technique.

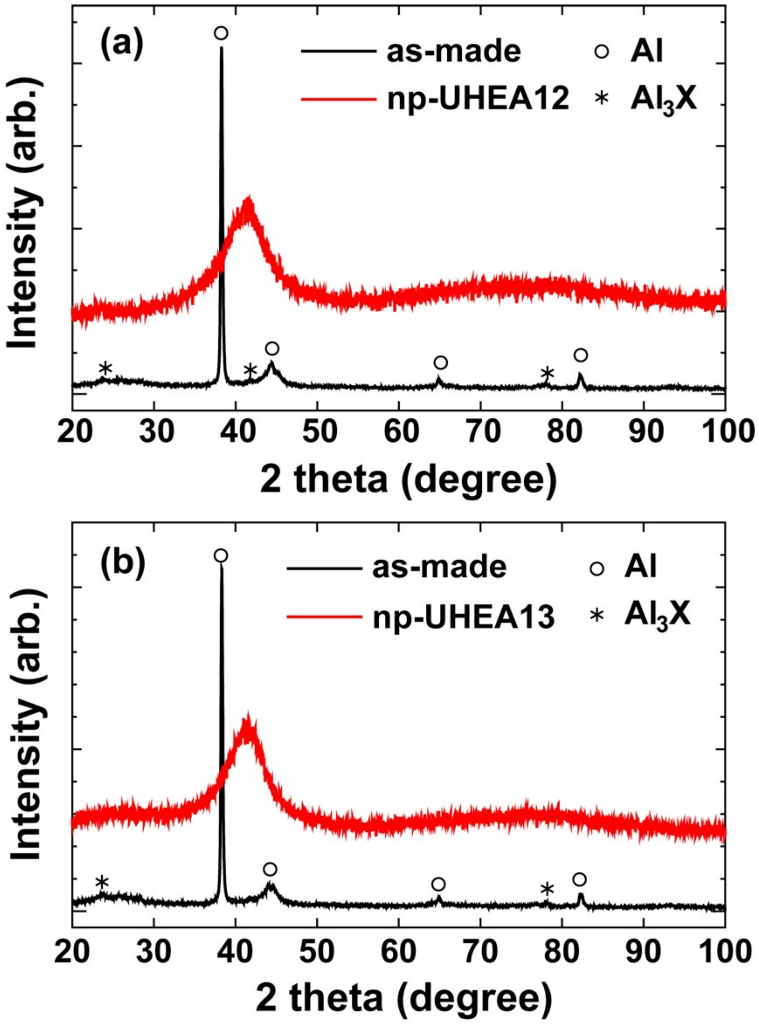

Figure S2. XRD patterns of (a) np-UHEA12 and as-made precursor alloy $Al_{89}Ag_1Au_1Co_1Cu_1Fe_1Ir_1Ni_1Pd_1Pt_1Rh_1Ru_1$ quenched by melt spinning, and (b) np-UHEA13 and $Al_{88}Ag_1Au_1Co_1Cu_1Fe_1Ir_1Mo_1Ni_1Pd_1Pt_1Rh_1Ru_1$ quenched by melt spinning.

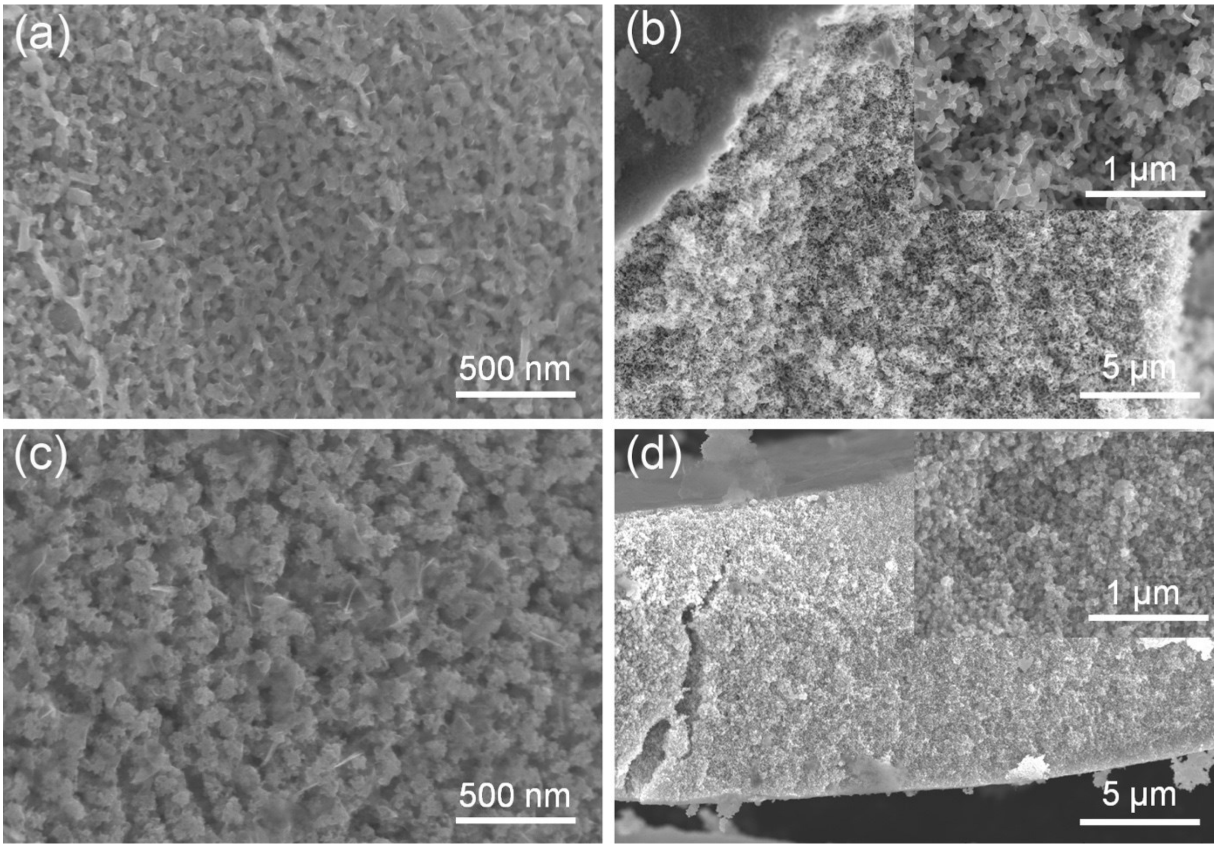

Figure S3. SEM images from (a, c) top-view and (b, d) cross-section regions of (a, b) np-UHEA12 and (c, d) np-UHEA13 showing the uniform nanoporous structure formed via dealloying.

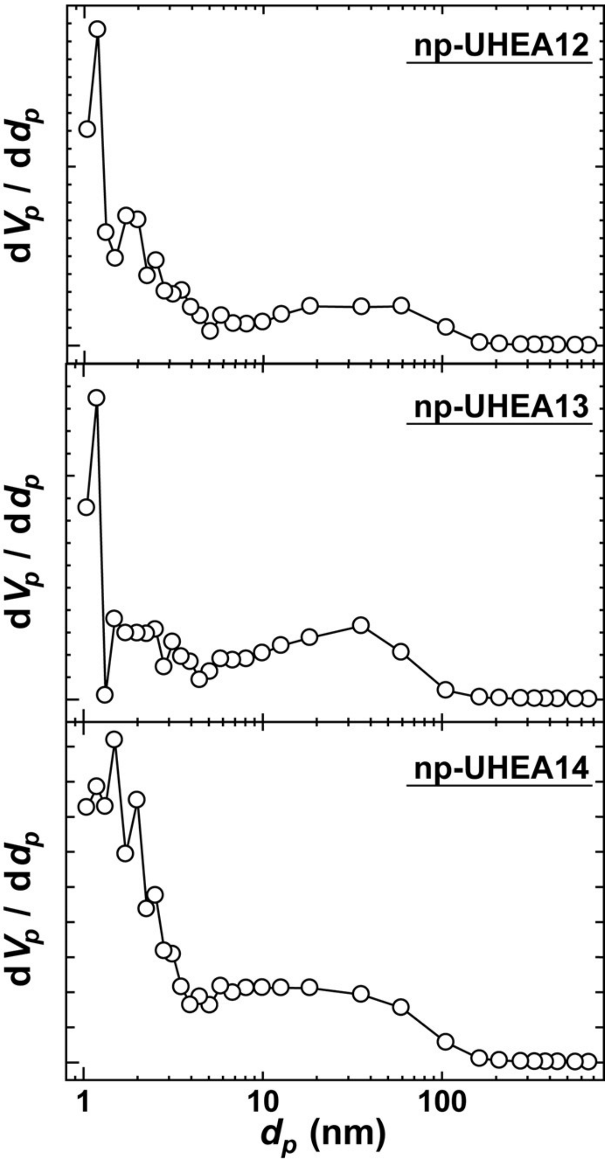

Figure S4. BJH pore size distributions of np-UHEA12, np-UHEA13, and np-UHEA14 showing similar hierarchical distributions with a high population of ~2–5 nm-sized particles and a broad peak for ~30–40 nm particles, consistent with the TEM observations.

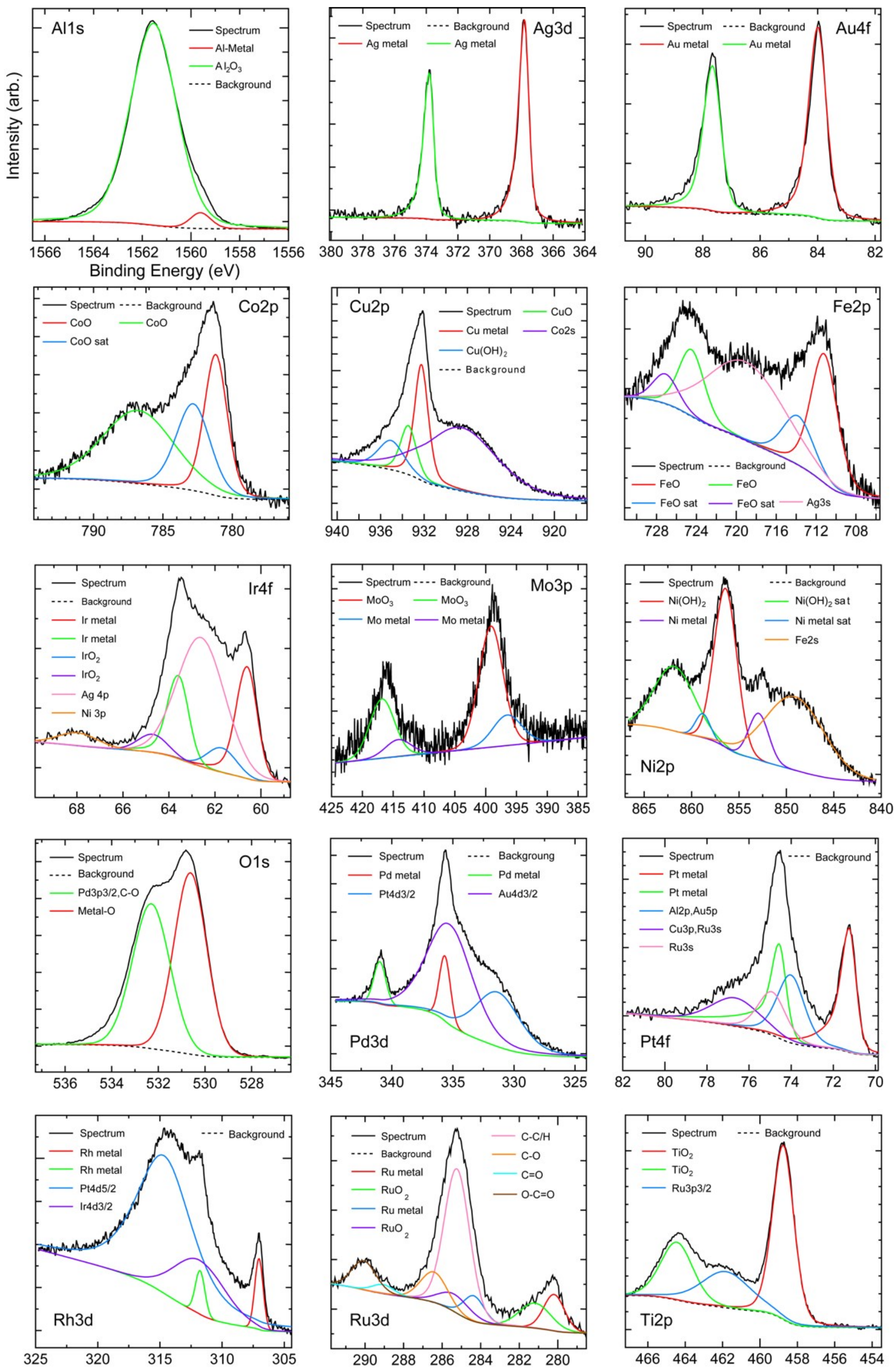

Figure S5. XPS spectra and deconvoluted states for each element in np-UHEA14.

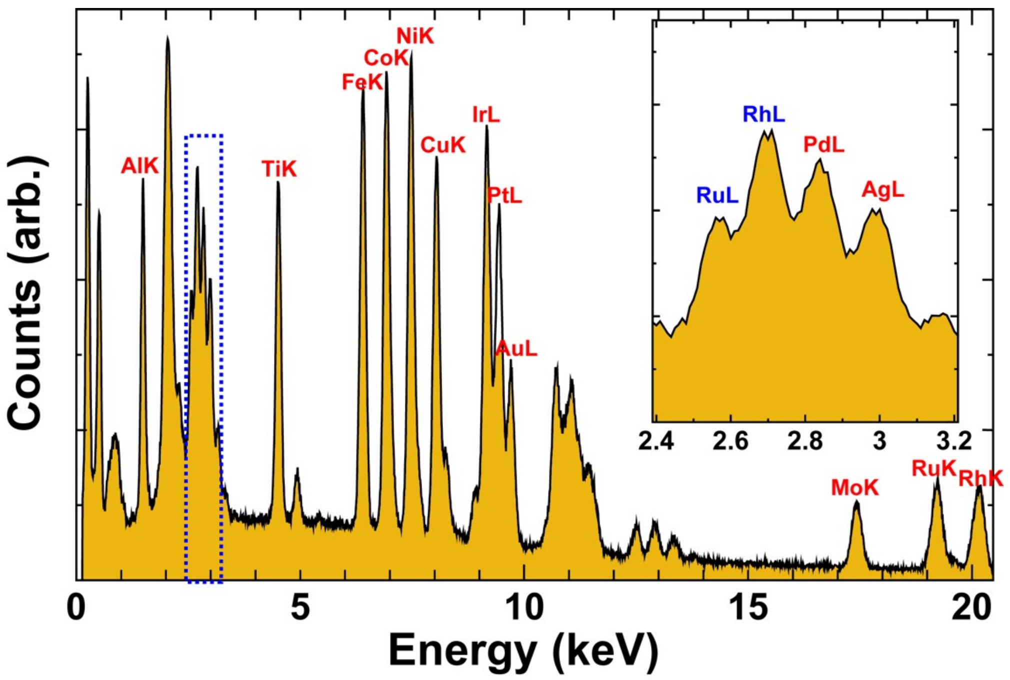

Figure S6. Typical EDS spectrum acquired for np-UHEA14. The selected ROI is also displayed for clarification. The 14 marked lines in red were used to display EDS mapping. A carbon-coated nylon mesh without metal elements was used to suppress the background intensities.

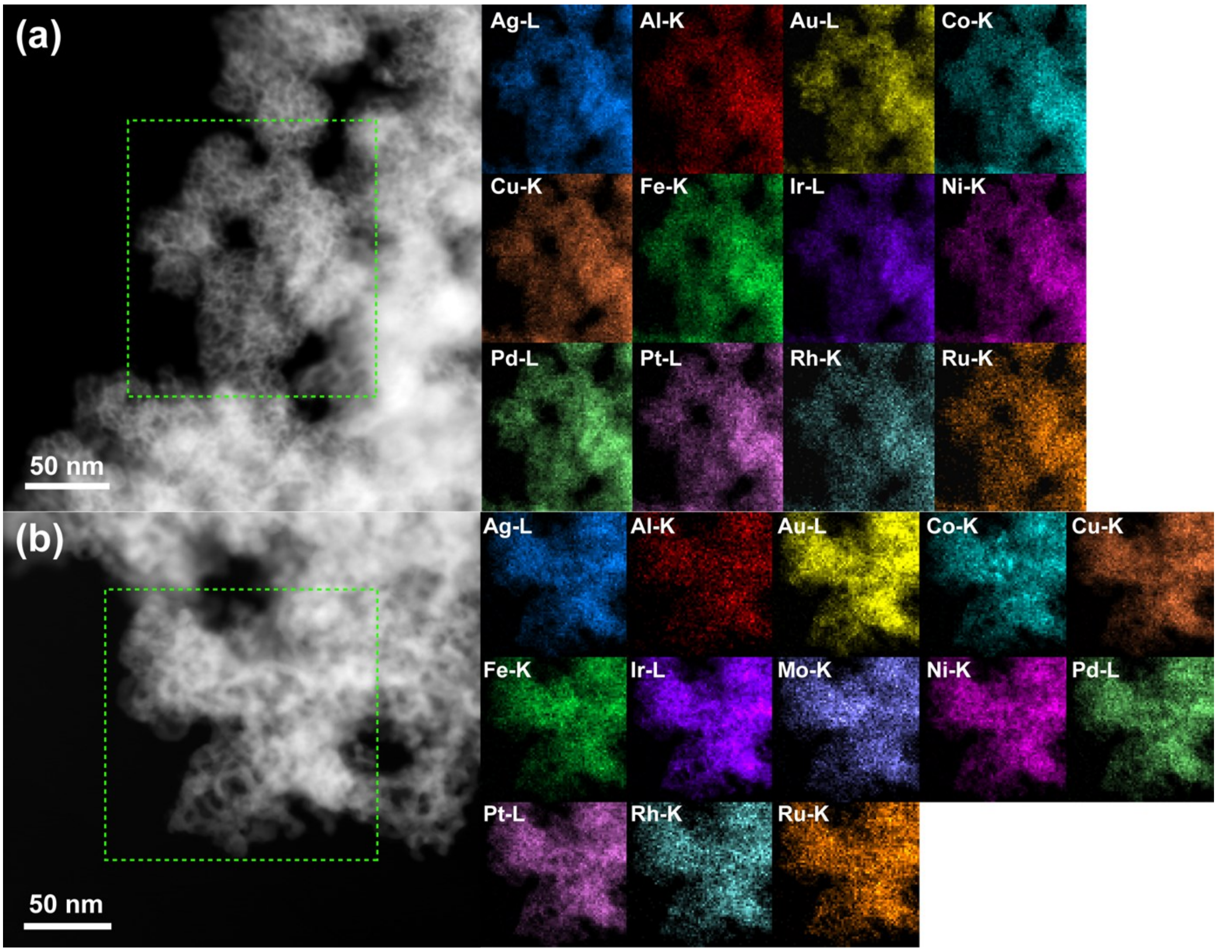

Figure S7. STEM-EDS mapping of (a) np-UHEA12 and (b) np-UHEA13. Selected ROIs are shown. Each element is uniformly distributed in both samples.

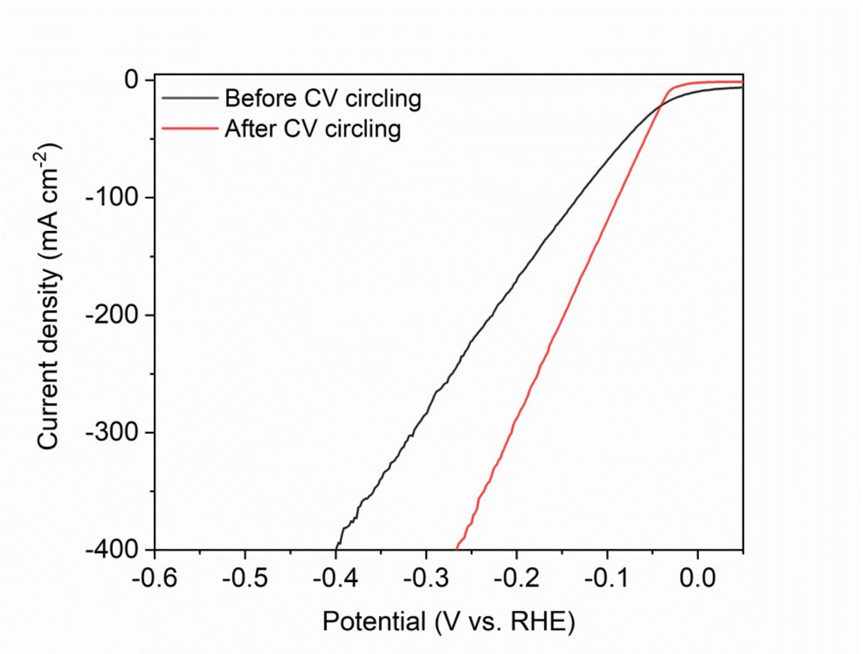

Figure S8. The HER properties of np-UHEA14 before and after CV circling at a sweep rate of 100 mV s^{-1} in the range of 1.1– 1.6 V vs. the reversible hydrogen electrode (RHE) in the electrolyte.

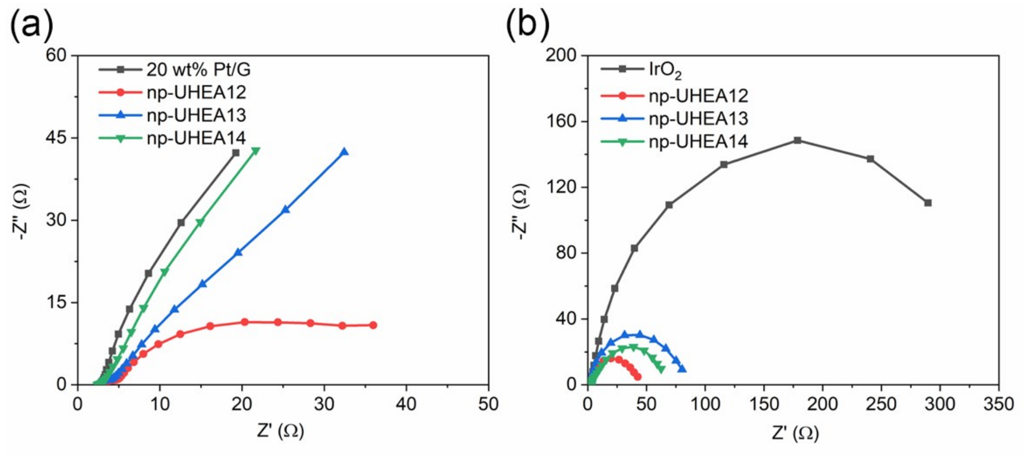

Figure S9. Nyquist plots of np-UHEAs, Pt/G, and IrO_2 collected at (a) 0.0 V vs. RHE and (b) at 1.5 V vs. RHE in 0.5 M H_2SO_4.

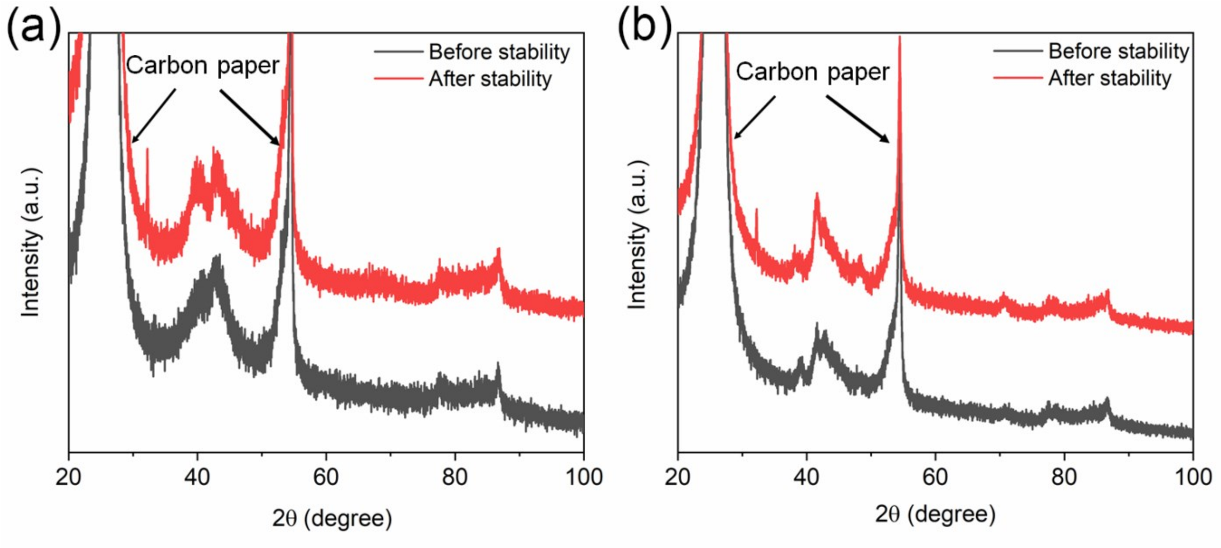

Figure S10. Comparation of XRD spectra of samples before and after the stability test in 0.5 M H_2SO_4, (a) np-UHEA-14 for HER, (b) np-UHEA-12 for OER.

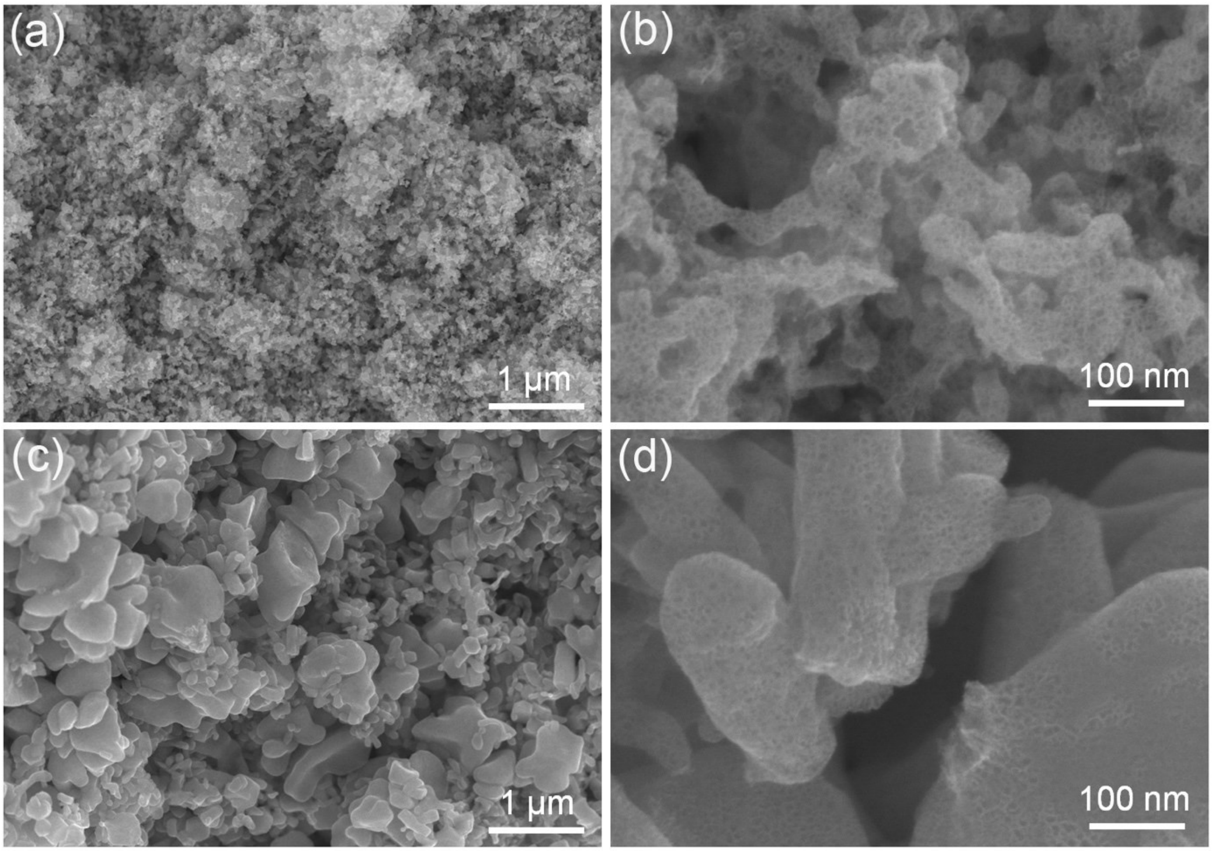

Figure S11. SEM images of np-UHEAs after 10 h of chronopotentiometric measurements at a current density of 10 mA cm^{-2} in 0.5 M H_2SO_4. (a, b) np-UHEA14 for HER and (c, d) np-UHEA12 for OER.

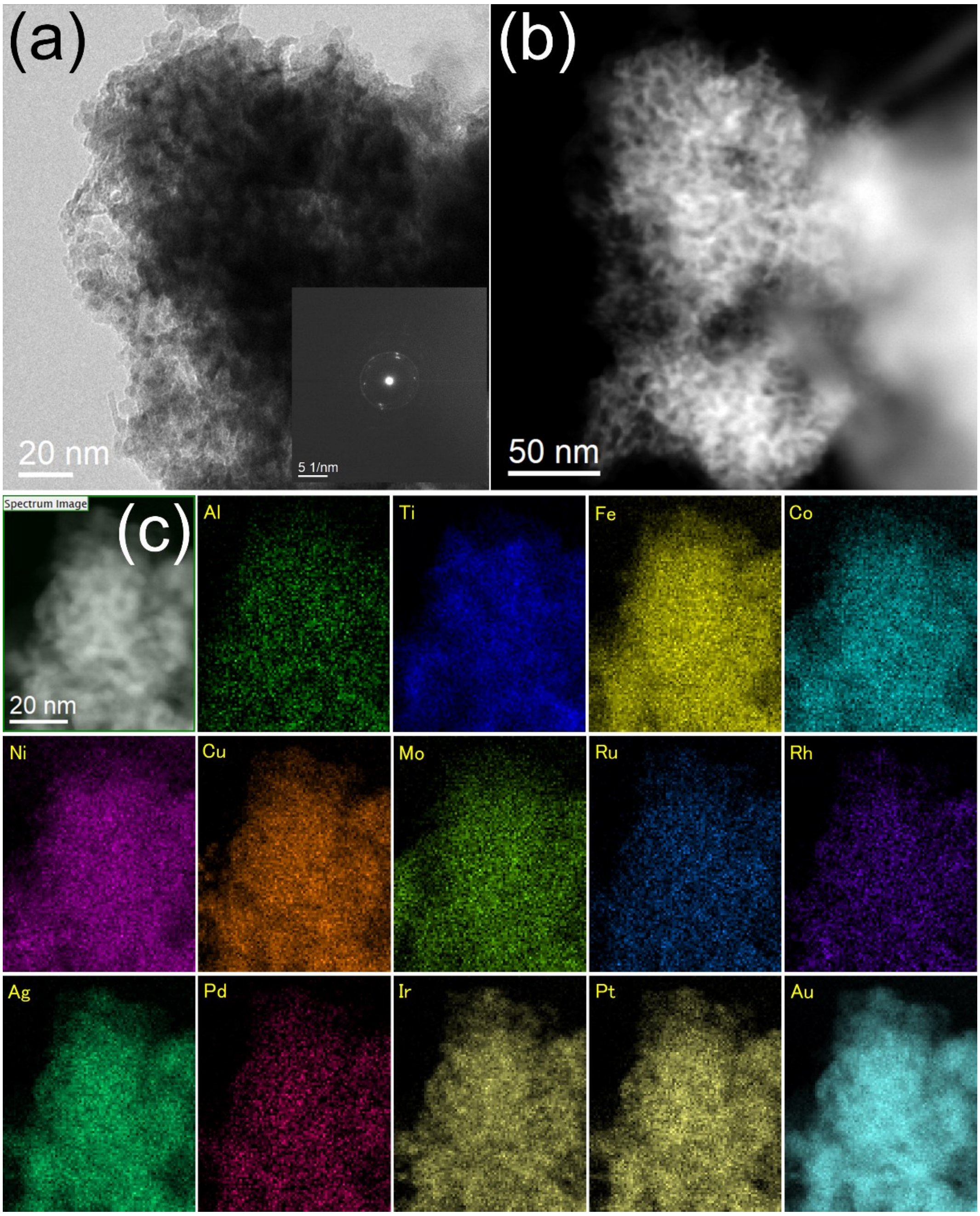

Figure S12. (a) TEM (inset SAED), (b) STEM, and (c) EDS mappings of np-UHEA14 after the stability test for HER in 0.5 M H_2SO_4. Each element remained uniformly distributed after the stability test.

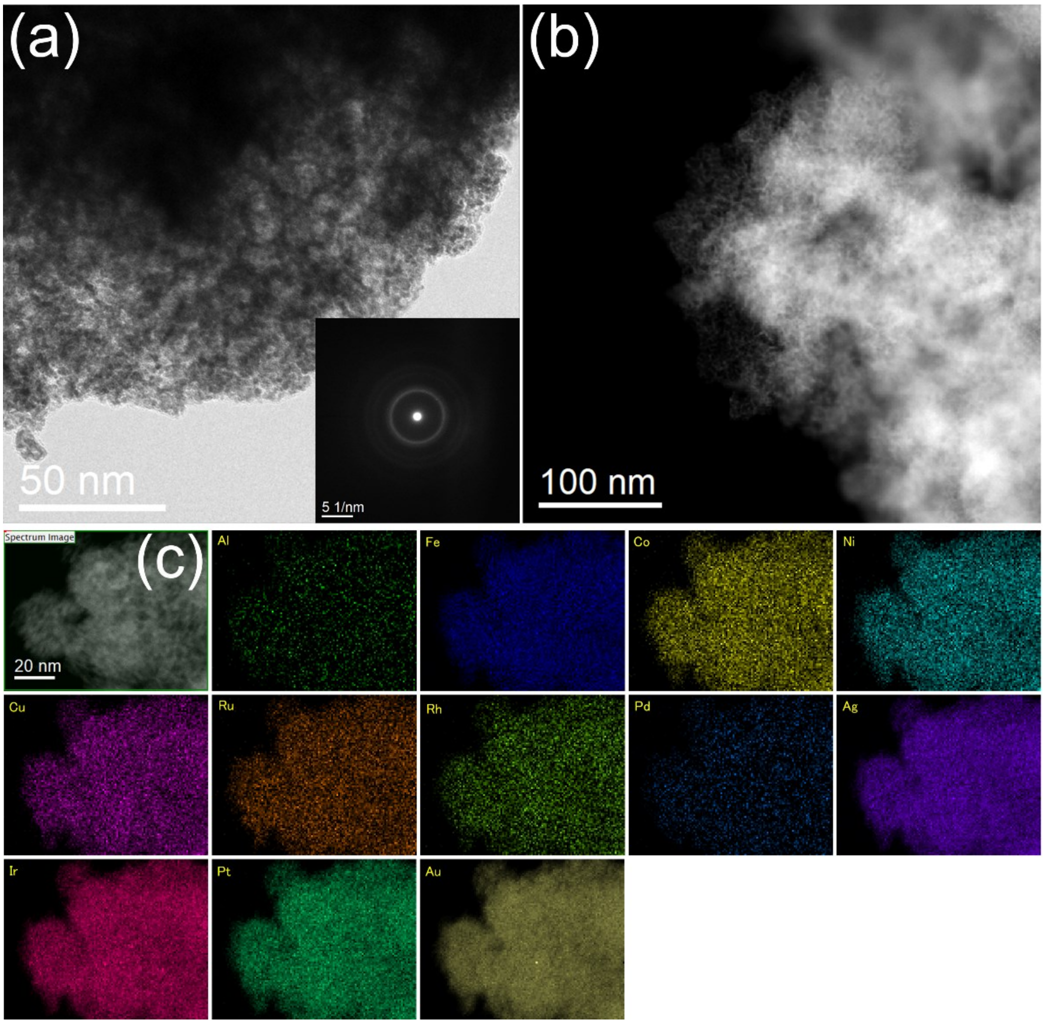

Figure S13. (a) TEM (inset SAED), (b) STEM, and (c) EDS mappings of np-UHEA12 after stability test for OER in 0.5 M H_2SO_4. Each element remained uniformly distributed after the stability test.

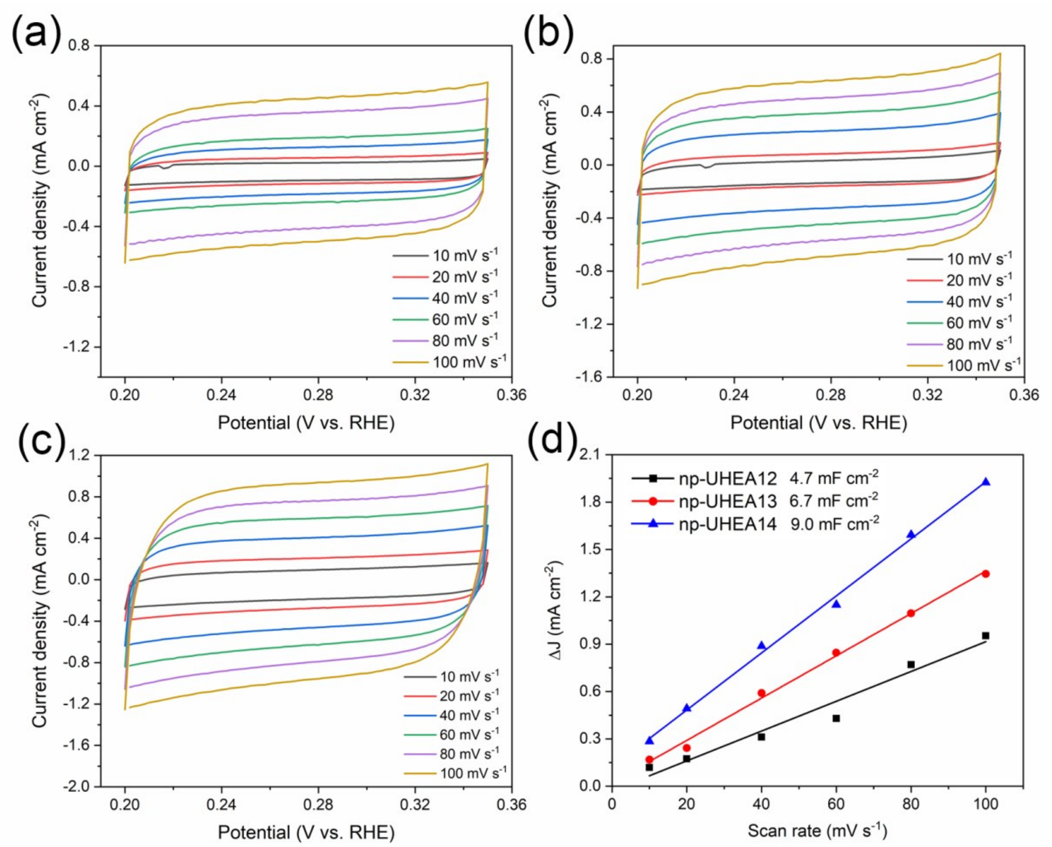

Figure S14. Charging currents measured in the non-faradaic potential range of 0.20–0.35 V (vs. RHE) at varying scan rates (10, 20, 40, 60, 80, and 100 mV s^{-1}) for (a) np-UHEA12, (b) np-UHEA13, and (c) np-UHEA14. (d) Charging currents measured at 0.25 V vs. RHE plotted as a function of the scan rate. The double layer capacitance of the system is calculated from the slopes of the linear fit to the data.

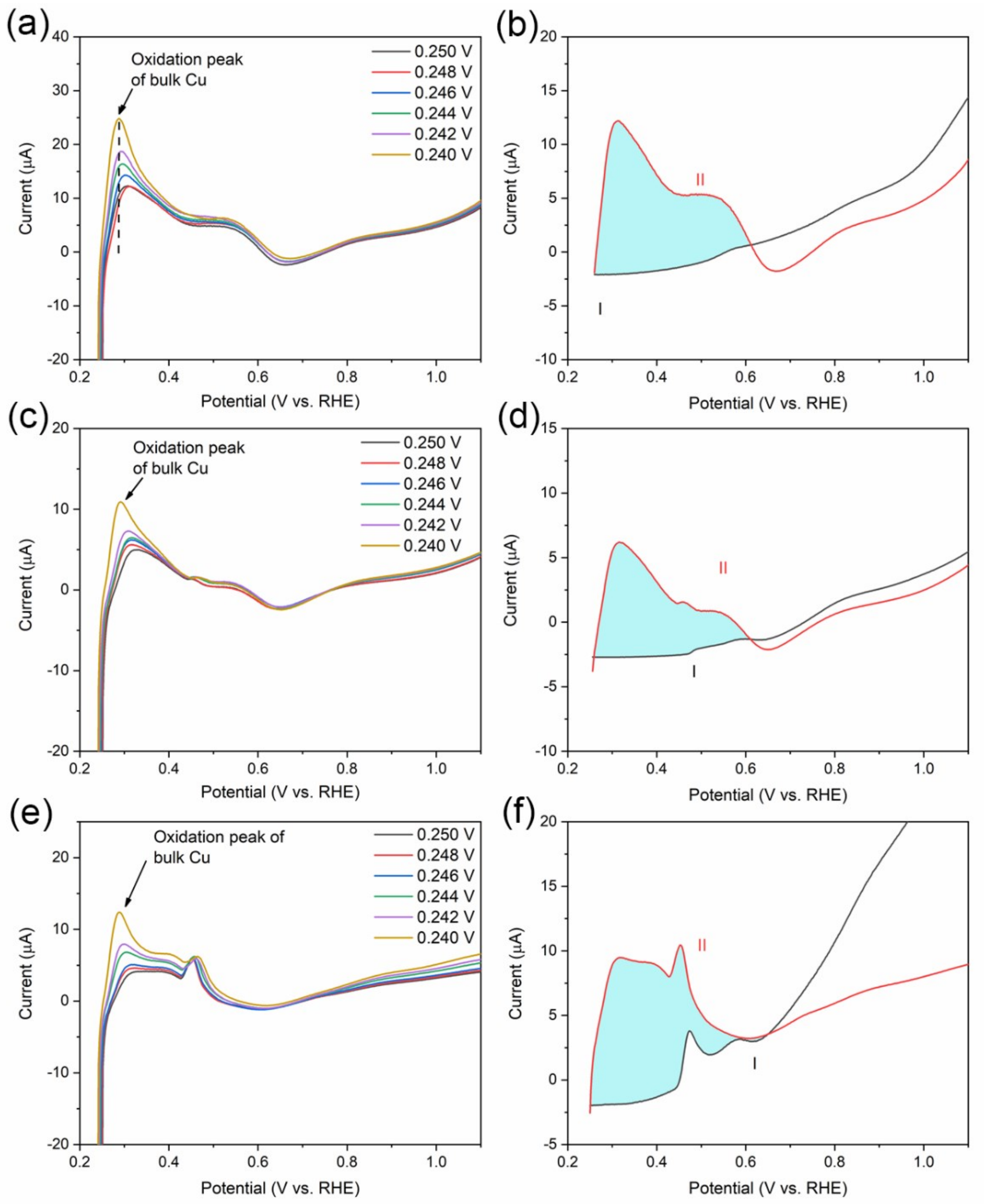

Figure S15. Electrochemical surface area (ECSA) measurements of np-UHEAs. Cu-UPD in 0.5 M H_2SO_4 in the presence of 5 mM $CuSO_4$ on (a) np-HEA, (c) np-UHEA13, and (e) UHEA14, the electrode was polarized at varying potentials for 100 s to form the UPD layers. Cu-UPD in 0.5 M H_2SO_4 in the (I) absence and (II) presence of 5 mM $CuSO_4$ on (b) np-UHEA12, (d) np-UHEA13, and (f) np-UHEA14 catalysts.

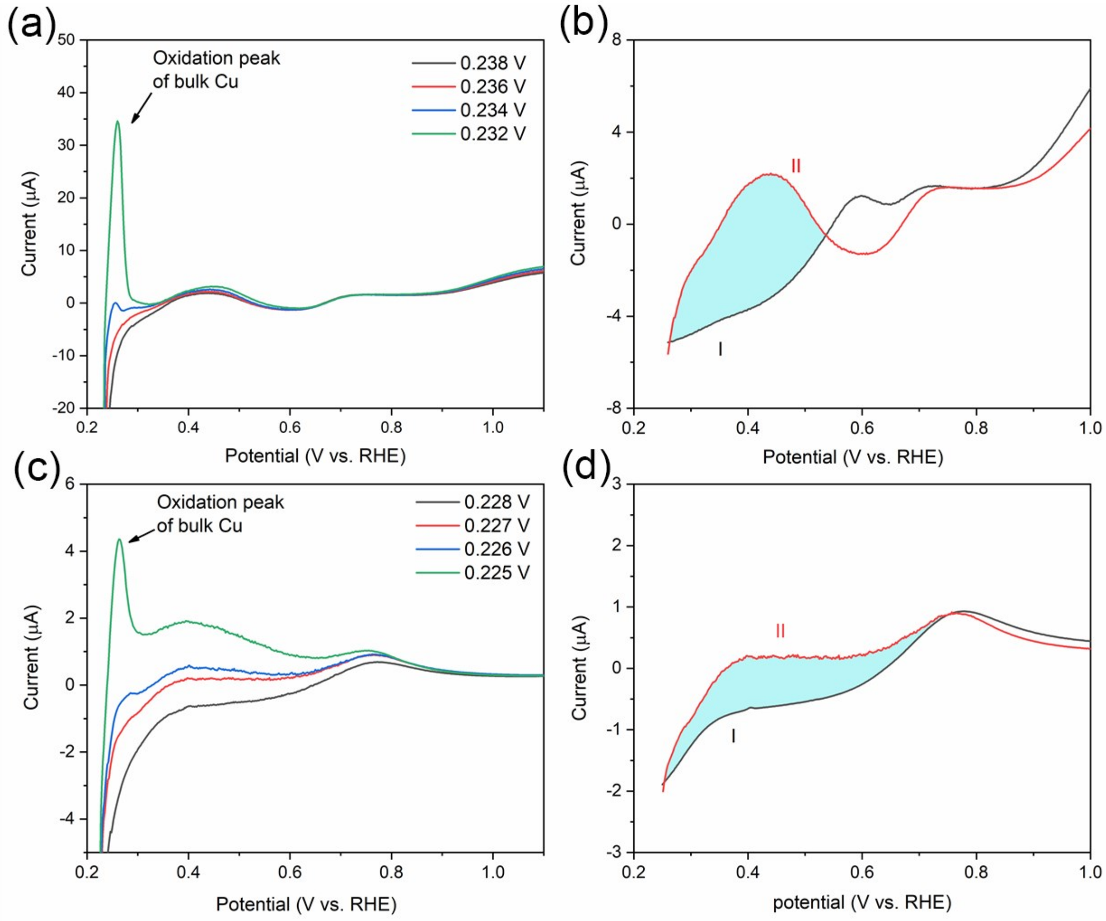

Figure S16. ECSA measurements of commercial Pt/G and IrO_2 catalysts. Cu-UPD in 0.5 M H_2SO_4 in the presence of 5 mM $CuSO_4$ on (a) commercial Pt/G and (c) commercial IrO_2. The electrode was polarized at varying potentials for 100 s to form the UPD layers. Cu-UPD in 0.5 M H_2SO_4 in the (I) absence and (II) presence of 5 mM $CuSO_4$ on (b) commercial Pt/G and (d) commercial IrO_2 catalysts.

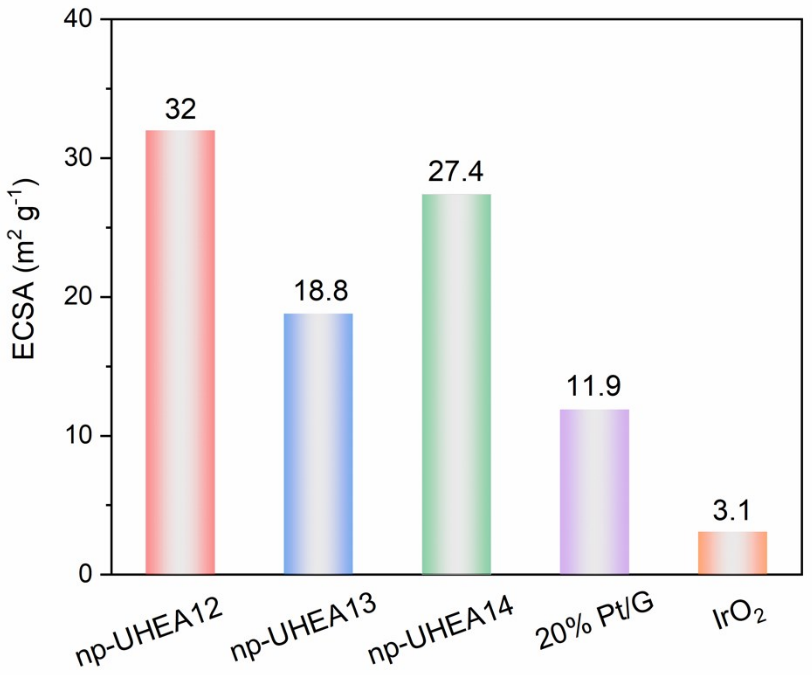

Figure S17. ECSAs of the catalysts measured employing the Cu-UPD method.

Table 1. Summary of electrocatalytic OER and HER performances of HEA-based catalysts.

Reaction	Catalyst name	Number of compositions	Electrolyte	Overpotential (mV @10 mA cm^{-2})	Tafel slope (mV dec^{-1})	Mass activity (A mg^{-1})	Durability test	Ref.
OER	np-UHEA12	12	0.5 M H_2SO_4	258	84.2		40,000 s @ 10 mA cm^{-2}	This work
	np-AlNiCoIrMo	5	0.5 M H_2SO_4	233	55.2	0.115 @1.5 V_{RHE}	11.5 mV increased @ 60 mA cm^{-2} after 7,000 cycles	S1
	np-AlNiCoIrV	5	0.5 M H_2SO_4		59.8			S1
	np-MoNiCoFeAl	5	1.0 M KOH	~240	46		~90% after 2,000 cycles, 50 h @ 10 mA cm^{-2}	S2
	CoCrFeMnNi NPs	5	0.1 M NaOH		150	0.104 @ 1.7 V_{RHE} including carbon black		S3
	PtLaNiCoFe HEMG-NP	5	0.1 M KOH	377	150		1 h @ 1.6 V_{RHE}	S4
	HEAN@NPC/CC-450	5	1.0 M KOH	263	43		24 h @ 10 mA cm^{-2}	S5
	Graphene-FeCoNiCuCr HEA nanocomposite	5	1.0 M KOH	330	80	380 mA mg^{-1} @ 1.75 V_{RHE}	7 h @ 25 mA cm^{-2}	S6
	AlNiCoRuMo nanowires	5	1.0 M KOH	245	54.5	~300 mA mg^{-1}_{Ru} @ 1.5 V_{RHE}	100 h @1.48 V_{RHE}	S7
HER	np-UHEA14	14	0.5 M H_2SO_4	32	30.1	2.44 A mg^{-1}_{Pt}	40,000 s @ 10 mA cm^{-2}	This work
	np-IrMoNiCoAl	5	0.5 M H_2SO_4	18.5	33.2		~ 50 h @ 1.52 V_{RHE}	S1
	np-AuPtPdNiCuAl	6	0.5 M H_2SO_4		28	6.9 @ -0.07 V_{RHE} 2.5 A $mg_{Pt+Pd+Au}^{-1}$ @ -0.07 V_{RHE}	~ 100% after 2,000 cycles	S8
	MoNiCoFeCr-1150	5	0.5 M H_2SO_4	107	41		8 h @ 100 mA cm^{-2}	S9
			1.0 M KOH	172	66			
	$Al_{80}Ni_6Co_3Mn_3Y_5Au_3$	6	0.5 M H_2SO_4	70	39		20 h @10 mA cm^{-2}	S10
	AuPtPdRhRu NPs/C-700 °C	5	1.0 M KOH	190 @ 30 mA cm^{-2}	62		8 h @ 100 mA cm^{-2}	S11
	PdFeCoNiCu/C	5	1.0 M KOH	18	39	6.51 A mg_{Pd}^{-1} @ -0.07 V_{RHE}	15 days @ 18 mV	S12
	FeCoPdIrPt@GO HEA-NPs	5	1.0 M KOH	42	82	9.1 mA μg_{Pt} @ 100 mV_{RHE}	150 h @ 10 mA cm^{-2}	S13
	$Pt_{18}Ni_{26}Fe_{15}Co_{14}Cu_{27}$/C	5	1.0 M KOH	11	30	10.96 A mg^{-1}_{Pt} @ -0.07 V_{RHE}	~ 100% after 10,000 cycles	S14
	AlNiCoRuMo nanowires	5	1.0 M KOH	24.5	30.3	~230 mA mg^{-1}_{Ru} @ −0.025 V	100 h @ -0.025 V_{RHE}	S7

IrPdPtRhRu HEA NPs	5	1.0 M KOH	17			~ 100% after 3,000 cycles	S15
FeCoNiAlTi HEI	5	1.0 M KOH	88.2	40.1		40 h @ 100 mA cm^{-2}	S16

Table S2. Comparison of the performances of bifunctional electrocatalysts for overall water splitting in acidic environments.

Catalysts	Electrolytes	HER η (mV @ 10 mA cm^{-2})	OER η (mV @ 10 mA cm^{-2})	Overall water splitting cell voltage (V@ 10 mA cm^{-2})	Durability test of water splitting.	Ref.
More than 12 elements np-UHEAs	0.5 M H_2SO_4	32 (np-UHEA14)	258 (np-UHEA12)	1.522	40,000 s @ 10 mA cm^{-2}	This work
np-AlNiCoIrMo	0.5 M H_2SO_4	18.5	233	1.48	48 h @ 1.52 V	[S1]
Ir-SA@Fe@NCNT	0.5 M H_2SO_4	26	250	1.51	12 h @ 1.51 V	[S17]
M-doped RuO_2 NWs (M = Co, Ni, Fe)	0.5 M H_2SO_4	78 (Ni-doped RuO_2 NWs)	200 (Ni-doped RuO_2 NWs)	1.537	12 h @ 1.537 V	[S18]
IrCoNi PHNCs	0.5 M H_2SO_4	68	309	1.6 @4.66 mA cm^{-2}	1000 CV circles	[S19]
Ir_6Ag_9 NTs	0.5 M H_2SO_4	20	285	1.55	1000 CV cycles	[S20]
$IrNi_{0.57}Fe_{0.82}$ NPs	0.5 M $HClO_4$	24	284	1.64	20000 s @ 10 mA cm^{-2}	[S21]
Ir/GF	0.5 M H_2SO_4	7	290	1.55	10 h @ 10 mA cm^{-2}	[S22]
IrCoNi PHNCs	0.5 M H_2SO_4	68	309	1.56 @2 mA cm^{-2}	1000 CV cycles	[S23]
RuCu NSs/C	0.5 M H_2SO_4	19	236	1.49	14 h @ 5 mA cm^{-2}	[S24]
Li-$IrSe_2$	0.5 M H_2SO_4	55	220	1.44	24 h @ 20 mA cm^{-2}	[S25]
IrNi NFs	0.5 M H_2SO_4	40	330	1.6	1,000 CV cycles	[S26]
Ru-SA/$Ti_3C_2T_x$	0.1 M $HClO_4$	70	290	1.56	32 h @ 10 mA cm^{-2}	[S27]
Co-RuIr	0.1 M $HClO_4$	24	235	1.52	24 h @ 10 mA cm^{-2}	[S28]
$IrCo_{0.65}$ NDs	0.1 M $HClO_4$	17	281	1.593	20,000 s @ 10 mA cm^{-2}	[S29]
PdCu/Ir/C	0.5 M H_2SO_4	21	344	1.583	2,000 CV cycles	[S30]

Comment on nominal composition of np-UHEAs

Acquiring the nominal composition of the np-UHEAs via ICP analysis was challenging because the samples were no completely soluble, even in aqua regia, and some substances remained undissolved as alloys or oxides. Here, we list the weight fractions of the measured elements without the unavailable elements Ag, Ir, Rh, and Ru, as is. We acknowledge that the sum of all weight percentages is not 100%. Although we cannot exclude the possibility that undetected volume among measured elements may exist as unsolved substances in aqua regia, the values of weight percent/atomic weight, except for Al, were found to be largely equal. Therefore, we assumed the nominal composition of np-UHEAs as follows:

$Al_{23}Ag_{7}Au_{7}Co_{7}Cu_{7}Fe_{7}Ir_{7}Ni_{7}Pd_{7}Pt_{7}Rh_{7}Ru_{7}$,

$Al_{22}Ag_{6.5}Au_{6.5}Co_{6.5}Cu_{6.5}Fe_{6.5}Ir_{6.5}Mo_{6.5}Ni_{6.5}Pd_{6.5}Pt_{6.5}Rh_{6.5}Ru_{6.5}$, and

$Al_{20.7}Ag_{6.1}Au_{6.1}Co_{6.1}Cu_{6.1}Fe_{6.1}Ir_{6.1}Mo_{6.1}Ni_{6.1}Pd_{6.1}Pt_{6.1}Rh_{6.1}Ru_{6.1}Ti_{6.1}$ (%) for np-UHEA12, np-UHEA13, and np-UHEA14, respectively. The corresponding mixed configuration entropies $S = -R \ \Sigma x_i \ln(x_i)$ were 2.39, 2.47, and 2.54, respectively.

Table S3. ICP analysis of np-UHEA12.

Element	Weight percent	Weight percent/ atomic weight
**Al**	5.6	0.237591
**Au**	11.6	0.067416
**Cu**	3.8	0.068457
**Co**	3.6	0.069928
**Fe**	3.4	0.069697
**Ni**	3.4	0.066313
**Pd**	6.3	0.067764
**Pt**	11.9	0.069825

Table S4. ICP analysis of np-UHEA13.

Element	Weight percent	Weight percent/ atomic weight
**Al**	5.9	0.218681
**Au**	11.3	0.057372
**Cu**	3.7	0.058231
**Co**	3.3	0.055999
**Fe**	3.1	0.055516
**Mo**	2.6	0.027095
**Ni**	3.4	0.057932
**Pd**	6.1	0.05732
**Pt**	11.2	0.057411

Table S5. ICP analysis of np-UHEA14.

Element	Weight percent	Weight percent/ atomic weight
**Al**	4.9	0.181616
**Au**	10.3	0.052295
**Cu**	3.4	0.05351
**Co**	3	0.050908
**Fe**	2.6	0.046562
**Mo**	3.8	0.0396
**Ni**	3	0.051116
**Pd**	5.6	0.052622
**Pt**	9.8	0.050235
**Ti**	2.5	0.052228

References

[S1] Z. Jin, J. Lv, H. Jia, W. Liu, H. Li, Z. Chen, X. Lin, G. Xie, X. Liu, S. Sun, H. J. Qiu, *Small* **2019**, *15*, 1904180.

[S2] H.-J. Qiu, G. Fang, J. Gao, Y. Wen, J. Lv, H. Li, G. Xie, X. Liu, S. Sun, *ACS Mater. Lett.* **2019**, *1*, 526.

[S3] F. Waag, Y. Li, A. R. Ziefuß, E. Bertin, M. Kamp, V. Duppel, G. Marzun, L. Kienle, S. Barcikowski, B. Gökce, *RSC Adv.* **2019**, *9*, 18547.

[S4] M. W. Glasscott, A. D. Pendergast, S. Goines, A. R. Bishop, A. T. Hoang, C. Renault, J. E. Dick, *Nat. Commun.* **2019**, *10*, 2650.

[S5] K. Huang, B. Zhang, J. Wu, T. Zhang, D. Peng, X. Cao, Z. Zhang, Z. Li, Y. Z. Huang, *J. Mater. Chem. A* **2020**, *8*, 11938.

[S6] R. Nandan, M. Y. Rekha, H. R. Devi, C. Srivastavab, K. K. Nanda, *Chem Commun.* **2021**, DOI: 10.1039/d0cc06485h.

[S7] Z. Jin, J. Lyu, Y.-L. Zhao, H. Li, X. Lin, G. Xie, X. Liu, J.-J. Kai, H.-J. Qiu, *ACS Mater. Lett.* **2020**, *2*, 1698.

[S8] H. J. Qiu, G. Fang, Y. Wen, P. Liu, G. Xie, X. Liu, S. Sun, *J. Mater. Chem. A* **2019**, *7*, 6499.

[S9] G. Zhang, K. Ming, J. Kang, Q. Huang, Z. Zhang, X. Zheng, X. Bi, *Electrochim. Acta* **2018**, *279*, 19.

[S10] S. Ju, J. Feng, P. Zou, W. Xu, J. Huo, S. Wang, W. Gao, H. Qiu, J. Wang, *J. Mater. Chem. A* **2020**, *8*, 3246.

[S11] M. Liu, Z. Zhang, F. Okejiri, S. Yang, S. Zhou, S. Dai, *Adv. Mater. Interfaces* **2019**, *6*, 1900015.

[S12] D. Zhang, Y. Shi, H. Zhao, W. Qi, X. Chen, T. Zhan, S. Li, B. Yang, M. Sun, J. Lai, B. Huang, L. Wang, *J. Mater. Chem. A* **2021**, DOI: 10.1039/D0TA10574K.

[S13] S. Gao, S. Hao, Z. Huang, Y. Yuan, S. Han, L. Lei, X. Zhang, R. Shahbazian-Yassar, J. Lu, *Nat. Commun.* **2020**, *11*, 2016.

[S14] H. Li, Y. Han, H. Zhao, W. Qi, D. Zhang, Y. Yu, W. Cai, S. Li, J. Lai, B. Huang, L. Wang, *Nat, Commun.* **2020**, *11*, 5437.

[S15] D. Wu, K. Kusada, T. Yamamoto, T. Toriyama, S. Matsumura, I. Gueye, O. Seo, J. Kim, S. Hiroi, O. Sakata, S. Kawaguchi, Y. Kubotag, H. Kitagawa, *Chem. Sci.* **2020**, *11*, 12731.

[S16] Z. Jia, T. Yang, L. Sun, Y. Zhao, W. Li, J. Luan, F. Lyu, L.-C. Zhang, J. J. Kruzic, J.-J. Kai, J. C. Huang, J. Lu, C. T. Liu, *Adv. Mater.* **2020**, *32*, 2000385.

[S17] F. Luo, H. Hu, X. Zhao, Z. Yang, Q. Zhang, J. Xu, T. Kaneko, Y. Yoshida, C. Zhu, W. Cai, *Nano Lett.* **2020**, *20*, 2120.

[S18] J. Wang, Y. Ji, R. Yin, Y. Li, Q. Shao, X. Huang, *J. Mater. Chem. A* **2019**, *7*, 6411.

[S19] J. Feng, F. Lv, W. Zhang, P. Li, K. Wang, C. Yang, B. Wang, Y. Yang, Ji. Zhou, F. Lin, G.‐C. Wang, S. Guo, *Adv. Mater.* **2017**, *29*, 1703798.

[S20] M. Zhu, Q. Shao, Y. Qian, X. Huang, *Nano Energy* **2019**, *56*, 330.

[S21] L. Fu, G. Cheng, W. Luo, *J. Mater. Chem. A* **2017**, *5*, 24836.

[S22]J. Zhang, G. Wang, Z. Liao, P. Zhang, F. Wang, X. Zhuang, E. Zschech, X. Feng, *Nano Energy* **2017**, *40*, 27–3328.

[S23] J. Feng, F. Lv, W. Zhang, P. Li, K. Wang, C. Yang, B. Wang, Y. Yang, J. Zhou, F. Lin, G.‐C. Wang, S. Guo, *Adv. Mater.* **2017**, *29*, 1703798.

[S24] Q. Yao, B. Huang, N. Zhang, M. Sun, Q. Shao, X. Huang, *Angew. Chem. Int. Ed.* **2019**, *58*, 13983.

[S25] T. Zheng, C. Shang, Z. He, X. Wang, C. Cao, H. Li, R. Si, B. Pan, S. Zhou, J. Zeng, *Angew. Chem. Int. Ed.* **2019**, *131*, 14906.

[S26] F. Lv, W. Zhang, W. Yang, J. Feng, K. Wang, J. Zhou, P. Zhou, S. Guo, *Small Methods* **2020**, *4*, 1900129.

[S27] X. Peng, S. Zhao, Y. Mi, L. Han, X. Liu, D. Qi, J. Sun, Y. Liu, H. Bao, L. Zhuo, H. L. Xin, J. Luo, X. Sun, *Small* **2020**, *16*, 2002888.

[S28] J. Shan, T. Ling, K. Davey, Y. Zheng, S.‐Z. Qiao, *Adv. Mater.* **2019**, *31*, 1900510.

[S29] L. Fu, X. Zeng, G. Cheng, W. Luo, *ACS Appl. Mater. Interfaces* **2018**, *10*, 24993.

[S30] M. Li, Z. Zhao, Z. Xia, M. Luo, Q. Zhang, Y. Qin, L. Tao, K. Yin, Y. Chao, L. Gu, W. Yang, Y. Yu, G. Lu, S. Guo, *Angew. Chem. Int. Ed.* DOI: 10.1002/anie.202016199.
